# Sperm Motility Annotated Genes: Are They Associated with Impaired Fecundity?

**DOI:** 10.3390/cells12091239

**Published:** 2023-04-25

**Authors:** Masood Abu-Halima, Lea Simone Becker, Mohammad A. Al Smadi, Hashim Abdul-Khaliq, Markus Raeschle, Eckart Meese

**Affiliations:** 1 Institute of Human Genetics, Saarland University, 66421 Homburg, Germany; 2 Department of Pediatric Cardiology, Saarland University Medical Center, 66421 Homburg, Germany; 3 Reproductive Endocrinology and IVF Unit, King Hussein Medical Centre, Amman 11733, Jordan; 4 Department of Molecular Genetics, TU Kaiserslautern, 67653 Kaiserslautern, Germany

**Keywords:** RNA, proteome, sperm, oligoasthenozoospermia, male infertility

## Abstract

Sperm motility is a prerequisite for achieving pregnancy, and alterations in sperm motility, along with sperm count and morphology, are commonly observed in subfertile men. The aim of the study was to determine whether the expression level of genes annotated with the Gene Ontology (GO) term ‘sperm motility’ differed in sperm collected from healthy men and men diagnosed with oligoasthenozoospermia. Reverse transcription quantitative real-time PCR (RT-qPCR), quantitative mass spectrometry (LC-MS/MS), and enrichment analyses were used to validate a set of 132 genes in 198 men present at an infertility clinic. Out of the 132 studied sperm-motility-associated genes, 114 showed differentially expressed levels in oligoasthenozoospermic men compared to those of normozoospermic controls using an RT-qPCR analysis. Of these, 94 genes showed a significantly lower expression level, and 20 genes showed a significantly higher expression level. An MS analysis of sperm from an independent cohort of healthy and subfertile men identified 692 differentially expressed proteins, of which 512 were significantly lower and 180 were significantly higher in oligoasthenozoospermic men compared to those of the normozoospermic controls. Of the 58 gene products quantified with both techniques, 48 (82.75%) showed concordant regulation. Besides the sperm-motility-associated proteins, the unbiased proteomics approach uncovered several novel proteins whose expression levels were specifically altered in abnormal sperm samples. Among these deregulated proteins, there was a clear overrepresentation of annotation terms related to sperm integrity, the cytoskeleton, and energy-related metabolism, as well as human phenotypes related to spermatogenesis and sperm-related abnormalities. These findings suggest that many of these proteins may serve as diagnostic markers of male infertility. Our study reveals an extended number of sperm-motility-associated genes with altered expression levels in the sperm of men with oligoasthenozoospermia. These genes and/or proteins can be used in the future for better assessments of male factor infertility.

## 1. Introduction

Infertility is a multifaceted disorder affecting approximately 15% of couples, with a male factor being implicated in around half of these cases [1]. It is well known that genetic factors play a role in the development of male infertility [2,3]. Thus, the altered expression of a single gene or genes can result in various male-infertility-related phenotypes [2]. Accurately identifying these genes represents the initial basis for understanding the molecular mechanisms involved in idiopathic cases of male infertility. Different forms of sperm dysfunction can be distinguished, and abnormalities in the basic semen parameters can identify male fertility status. Sperm motility often plays a role in difficulties achieving pregnancy, and defects in genes associated with the sperm flagellum and/or motility result in a loss of one of the sperm’s key features, the ability to progress through the female reproductive tract to achieve fertilization [4]. Sperm motility, together with sperm count and morphology, is one of the basic sperm quality parameters routinely evaluated in any andrological check-up [4]. A reduced percentage of motile sperm in a semen sample is defined as ″asthenozoospermia″ and is one of the main seminal pathologies affecting fecundity, with a prevalence of up to 81% [5]. An andrological examination based on the World Health Organization (WHO) is usually performed to assess the basic sperm functions. These examinations provide a predictive indicator of the functional status of men’s ability to achieve pregnancy. Although andrological examinations are the initial step in the evaluation of infertile men, they provide no insights into the functional potential of the sperm to fertilize the oocyte [6].

Studies have demonstrated that the dysregulation of certain RNAs is highly correlated with basic semen parameters, notably, sperm motility and/or sperm dysfunction [7,8,9,10]. These studies analyzed messenger RNAs (mRNAs) as well as different classes of regulatory non-coding RNAs, including microRNAs (miRNAs) isolated from human sperm, seminal plasma, and testes. However, for the majority of these genes and their targets (i.e., miRNA targets), it remains unclear how they contribute to the production of viable sperm capable of achieving pregnancy [11]. Therefore, the diagnostic value of such genes needs to be further validated, and their precise role in spermatogenesis investigated. The Gene Ontology (GO) database groups genes based on their occurrence and function, including genes associated with spermatogenesis dysfunction, reduced sperm motility, failure in capacitation, and fertilization.

High-throughput techniques have provided a recent overview of genes and proteins that are downregulated and/or upregulated at various stages of sperm development [12,13]. While omics data can provide valuable insights into the molecular mechanisms underlying various biological processes, it is important to ensure that these data are not influenced by technical artifacts or errors that could lead to false conclusions. One common approach for validating omics data is to use targeted assays, such as reverse transcription quantitative real-time PCR (RT-qPCR), liquid chromatography/tandem mass spectrometry (LC-MS-MS), and/or Western blotting, to measure the expression levels of specific genes or proteins identified by the omics analysis. This can help to confirm the differential expression of specific targets and provide additional information on the functional relevance of these targets.

In this study, we aimed to evaluate and validate the expression levels of 132 genes listed in the Gene Ontology (GO) database in the categories of Homo sapiens sperm motility (GO:0097722) and flagellated sperm motility (GO:0030317) in human sperm as biomarkers for a better assessment of male factor infertility and/or sperm dysfunction. To achieve this goal, we employed the RT-qPCR platform to validate the 132 selected genes associated with sperm motility. Using an independent set of human sperm collected from normozoospermic and oligoasthenozoospermic individuals, we performed an unbiased proteomic analysis coupled with LC-MS-MS and bioinformatics to validate the RT-qPCR. These approaches (i.e., RT-qPCR and LC-MS-MS) led to the validation of a set of sperm-motility-related genes. These validated genes can be used in the future for a better assessment of male factor infertility.

## 2. Materials and Methods

### 2.1. Study Population and Sample Collection

The study population comprised 99 men who had failed to achieve conception after a period of 1–2 years and were referred to the Reproductive Endocrinology and IVF Unit at King Hussein Medical Centre in Amman, Jordan. The control group consisted of age-matched normozoospermic sperm donors (n = 99) without infertility diagnoses and with no apparent female factors that could be a possible cause of the couple’s inability to conceive. To eliminate the influence of age on gene expression analysis, controls and subfertile patients were age-matched (mean age ± standard deviation 35.49 ± 6.31 years vs. 34.70 ± 6.73 years, *p* = 3.91 × 10^−1^). Semen samples were divided into two groups based on WHO’s 2010 guidelines for primary semen parameters, including liquefaction time, volume, pH, viscosity, sperm count, motility, and morphology. These parameters defined the two tested subgroups, i.e., oligoasthenozoospermia and normozoospermia, and all included subjects exhibited normal sperm morphology (≥4%). The diagnosis of spermiogram was confirmed by at least two embryologists on ejaculated semen collected after 3–5 days of sexual abstinence. Men with a medical history of infertility-related risk factors and/or other known factors, including infections and genetic diseases, such as Y-chromosome microdeletions and other chromosomal abnormalities, were excluded from the study. The study complies with the Declaration of Helsinki and was approved by the Institutional Review Board (Ha 195/11/updated June 2021) of the Saarland Medical Association. Ethical guidelines were followed in conducting the research, with written informed consent obtained from patients and volunteers before the experiments.

### 2.2. Sperm Purification and RNA Extraction

Sperm were purified from semen samples using a discontinuous PureSperm^®^ density gradient (Nidacon) to separate mature sperm from somatic cells, round cells, and leukocytes, as previously described [14]. Total RNA was extracted from the purified sperm fraction using the miRNeasy Mini Kit on the QIAcube™ Robotic Workstation (Qiagen, Hilden, Germany), following the manufacturer’s protocol with minor modifications. Briefly, sperm were mixed with 700 µL of QIAzol Lysis Reagent (Qiagen), supplemented with dithiothreitol (DTT) (80 mM; Sigma-Aldrich, Laclede Avenue. St. Louis, MO, USA), and allowed to completely lyse for 30 min at room temperature (RT). A DNase I (Qiagen) treatment step was included during the isolation procedure to remove any residual genomic DNA contamination. The purified RNA samples were eluted in 20 μL of RNase/DNase-free water, and their quality and quantity were determined using both an Agilent 2100 Bioanalyzer (Agilent Technologies, Santa Clara, CA, USA) and a NanoDrop 2000 Spectrophotometer (Thermo Fisher Scientific, Inc., Waltham, MA, USA), respectively.

### 2.3. Selection of Target Genes

Using AmiGO [15] and ToppGene Suite [16] gene ontology enrichment algorithms, we retrieved all genes and gene products annotated to Homo sapiens sperm motility (GO:0097722) and flagellated sperm motility (GO:0030317). In total, 132 genes related to motility and/or movement in sperm were selected for validation by RT-qPCR, as shown in Table S1. These genes are also known to be expressed and/or involved in spermatogenesis and/or sperm function.

### 2.4. Reverse Transcription and Pre-Amplification

Complementary DNA (cDNA) was generated in 5 µL reactions by reverse transcription (RT) of 75 ng RNA using the Reverse Transcription Master Mix (Fluidigm Corporation, South San Francisco, CA, USA), as previously described [17]. After the RT reaction was completed, the reactions were used immediately for preamplification reactions with Preamp Master Mix (Fluidigm) according to the manufacturer’s protocol PN 100-5875 C1. Briefly, in a microcentrifuge tube, 1 μL of each Delta Gene assay (100 μM stock, Table S1) was mixed with DNA Suspension Buffer (10 mM Tris, pH 8.0, 0.1 mM EDTA (TEKnova, Hollister, CA, USA) to make a concentration of 500 nM for each assay with a total volume of 200 μL. In a new 96-well plate called “Preamp Plate,” 5 µL mix containing 1.25 µL cDNA from each sample, 1 µL of the Preamp Master Mix, 0.5 µL Pooled Delta Gene assay mix (500 nM), and 2.25 µL DNase-free water were pipetted into each of the 96-well plates using a QIAcube^™^ Robotic Workstation (Qiagen). The pre-amplification of cDNA was performed using a TProfessional Thermocycler (Biométra) with the following thermal cycling conditions: 95 °C for 2 min, followed by 14 cycles at 95 °C for 15 s, and 60 °C for 4 min. The pre-amplified cDNA was then cleaned up with Exonuclease I (New England BioLabs, Ipswich, MA, USA). A mixture of 2 µL Exo I containing 0.4 µL Exonuclease I (20 U/μL), 0.2 µL Exonuclease I Reaction Buffer, and 1.4 µL DNase-free water was added to each 5 μL preamplification reaction using the QIAcube^™^ Robotic Workstation. Then, the preamplification reaction and Exo I reactions were digested at 37 °C for 30 min and inactivated at 80 °C for 15 min using the TProfessional Thermocycler. Lastly, each sample was diluted with 30.5 µL DNA suspension buffer using the QIAcube^™^ Robotic Workstation and then stored at −20 °C for the Biomark^™^ HD RT-qPCR (Fluidigm).

### 2.5. RT-qPCR

Biomark^™^ HD with a 96.96 IFC was used for the RT-qPCR amplification, as previously described [17]. Briefly, for each sample, a 6 µL sample mix containing 3 µL of 2× SsoFast EvaGreen Supermix with low ROX (Bio-Rad Laboratories, Hercules, CA, USA), 0.3 µL of 20× DNA Binding Dye (Fluidigm), and 2.7 µL of the pre-amplified sample was prepared. A primer stock (100 μM combined forward and reverse primers) was prepared for each assay, and 0.3 µL of the stock was mixed with 3 µL of 2× Assay Loading Reagent (Fluidigm) and 2.7 µL 1× DNA Suspension Buffer to make assay mixes. Finally, 5 μL of each assay and sample mix was transferred into the appropriate inlets according to Fluidigm’s recommendation. After loading, the array was placed in the Biomark HD instrument for quantification and detection using the GE Fast 96 × 96 PCR + Melt v2.pcl PCR thermal protocol. The data were analyzed with Real-Time PCR Analysis Software (Fluidigm) according to Fluidigm’s recommendation. A non-template cDNA/pre-amplification and a non-template pre-amplification control (H_2_O) were included and were finally defined as those with Ct values ≥ 35 or that were undetermined.

### 2.6. Protein Lysate Preparation and LC-MS/MS Analysis

Purified sperm samples were thawed on ice and washed three times with phosphate-buffered saline (PBS). These sperm samples were obtained from an independent cohort of 7 oligoasthenozoospermic men. Another independent cohort of 7 age-matched normozoospermic volunteers served as controls. One hundred microliters of lysis buffer (4% SDS, 100 mM Tris/HCl pH 7.6, 0.1 M DTT) was added to each sample, and samples were incubated at 95 °C for 5 min. Then, the samples were sonicated at 20 joules for 2 s × 10 at intervals of 10 s, incubated on ice for 30 min, and centrifuged at 14,000× *g* for 10 min, after which the supernatant was transferred to a new microcentrifuge tube. After that, an aliquot of protein lysate from each sample was subjected to enzymatic hydrolysis using the FASP (filter-aided sample preparation) enzymatic method as described by Wisniewski et al. [18] with a slight modification. Briefly, 90 µL of protein lysate was mixed in a Micro-con centrifugal filter unit (MRCF0R030, Merck Millipore, Burlington, MA, USA) with 600 µL of freshly prepared UA solution (8 M urea in 0.1 M Tris/HCl pH 8.5) and centrifuged at 14,000× *g* for 15 min at room temperature (RT) to remove SDS. The residual amount of SDS was removed by washing the centrifugal filter unit two times with 200 µL of UA at 14,000× *g* for 15 min at RT. Proteins were then alkylated with IAA solution (0.05 M iodoacetamide in UA) in the dark for 30 min at RT. Afterward, proteins were washed three times with 100 µL of UA solution, followed by three washes with 100 µL of ABC buffer (0.05 M NH4HCO3 in H_2_O). After each washing step, filters were centrifuged at 14,000× *g* for 15 min at RT. Finally, proteins were digested with 40 µL ABC containing trypsin (trypsin enzyme to protein ratio 1:100) overnight in a wet chamber at 37 °C for approximately 18 h. The next day, peptides were eluted from the centrifugal filter unit by centrifugation at 14,000× *g* for 15 min at RT, followed by the addition of 50 µL of 0.5 M NaCl and centrifugation at 14,000× *g* for 15 min at RT. Digested peptides were acidified with CF3COOH (trifluoroacetic acid, TFA, with a final concentration of 1%) and desalted on Empore C18 material according to Rappsilber et al. [19]. Lastly, peptides were eluted, dried using vacuum centrifugation, and reconstituted with 20 µL of 0.1% formic acid. Peptide concentrations were determined using the Pierce™ Quantitative Colorimetric Peptide Assay kit (Thermo Fisher Scientific).

### 2.7. Mass Spectrometry and Data Processing

Tryptic peptides were separated and analyzed directly on an EASY-nLC^™^ 1200 system (Thermo Fisher Scientific) chromatography system coupled to a Q Exactive HF-X Orbitrap LC-MS/MS System (Thermo Fisher Scientific) via a Nanospray ion source. Peptide separation was carried out in analytical columns (50 cm, 75 µm inner-diameter packed in-house with C18 resin ReproSilPur 120, 1.9 µm diameter) using a 3 h gradient at a flow rate of 250 nl/minute (Solvent A: aqueous 0.1% formic acid; Solvent B: 80% acetonitrile, 0.1% formic acid). Raw MS data files from single-shot experiments were processed with MaxQuant software (v1.6.3.3, Max Planck Institute of Biochemistry, Martinsried, Germany) using the human reference proteome database (UniProt: UP000005640). Raw data were normalized using the label-free quantitation (LFQ) algorithm implemented in MaxQuant. The exact parameters for chromatography, MS instrument settings, and data processing with MaxQuant can be retrieved from the raw files available at ProteomeXchange with the assigned accession number PXD039703, as referred to in Table S2. ProteomeXchange is a global initiative that promotes the sharing and exchange of proteomics data among researchers (www.proteomexchange.org).

### 2.8. Statistical and Bioinformatics Analysis

The statistical analysis was conducted using R software (v4.2.1, R Core Team, Vienna, Austria).

Continuous variables were described as means ± standard deviations. The unpaired two-tailed *t*-test was used to compare the differences between groups and variables. As for the RT-qPCR, the relative quantitative method was used to measure the dynamic change of gene expression levels [20]. Briefly, the ΔCt value is the difference between the Ct value of the gene of interest and the Ct value of the endogenous reference gene and was calculated using the equation: ΔCt = Ct ^(gene of interest)^ − Ct ^(endogenous reference gene)^. The unpaired two-tailed *t*-test was used to compare the differences between fertile and subfertile groups, and adjustment for multiple testing was performed by controlling the false discovery rate (FDR) according to the approach of Benjamini and Hochberg [20]. A volcano plot was generated based on log2 fold changes and adjusted *p*-values. The heatmaps were generated based on ΔCt values, and the rectangle in each heatmap plot represents the normalized expression level of the gene (the higher the value, the lower the expression level of the gene and vice versa), and negative values are related to the ΔCt values. As for MS measurements, LFQ normalized protein intensities were log2-transformed [20]. Protein groups were filtered to eliminate potential contaminants and reverse hits, and all proteins that were identified by site only were further considered. Protein groups were filtered to contain at least three valid values in at least one group (oligoasthenozoospermic men/normozoospermic controls). Missing values were imputed by values sampled from a normal distribution centered around the detection limit of the MS instrument with a width of 0.3 and a downshift of 1.8 relative to the standard deviation and mean of all protein intensities of the respective sample [21]. An unpaired two-tailed *t*-test was used to identify proteins with significant enrichment in the oligoasthenozoospermic men compared to those in the normozoospermic men. Adjusted *p*-values were calculated using the Benjamini–Hochberg procedure, and Log2 (fold change) of the mean LFQ intensities of oligoasthenozoospermic men compared to the normozoospermic men was determined. The volcano plot was generated based on Log2 (fold change) and adjusted *p*-values. The heatmaps were generated based on log2 LFQ intensities. The rectangle in each heatmap represents the normalized expression level of the protein (the higher the value, the higher the protein expression level). 

Heatmaps and volcano plots of differentially expressed genes/proteins were plotted using ggplot2 (version 3.3.6) and pheatmap (version 1.0.12) packages using R software. The R packages tidyverse (version 1.3.2), readxl (version 1.4.0), operators (version 0.1-8), naniar (version 0.6.1), ggrepel (version 0.9.1), viridis (version 0.6.2), and RColorBrewer (version 1.1-3) were additionally used. An overrepresentation analysis was carried out using only the genes and proteins that showed a significant expression level, and data were visualized using the enrichment algorithms implemented in the R packages ClusterProfiler (version 4.6.0), enrichplot (version 1.18.1), DOSE (version 3.24.2), and org.Hs.eg.db (version 3.16.0) of the Bioconductor Repository. 

Spearman’s correlation test was carried out to evaluate the correlation between the expression levels of genes associated with sperm motility, proteins, and basic semen parameters using the Hmisc (Version 4.7-1) R package.

## 3. Results

### 3.1. Basic Parameters of Oligoasthenozoospermic Men and Normozoospermic Controls

The characteristics of the spermiogram of the oligoasthenozoospermic samples (n = 99) and age-matched normozoospermic controls (n = 99) are shown in Table 1. Subfertile men with oligoasthenozoospermia were significantly different from normozoospermic men in terms of sperm count, motility, and morphology (*p* < 0.05). Other parameters, such as age, pH, and volume, were not significantly different.

### 3.2. Differentially Expressed Genes in Sperm (Determined by RT-qPCR Analysis)

The expression levels of 132 genes associated with sperm motility in the GO terms were evaluated in 92 men with oligoasthenozoospermia and 92 age-matched normozoospermic controls. Out of the 132 genes analyzed, 114 showed significantly different expression levels in the oligoasthenozoospermic men compared to normozoospermic controls (Table 2). Specifically, 94 genes showed a significantly lower expression level, and 20 genes showed a significantly higher expression level in the abnormal sperm samples, as shown in Figure S1. It was not possible to make a more detailed distinction between the oligoasthenozoospermic men and normozoospermic controls based on the hierarchical clustering of genes with the highest expression levels (log2 (fold change)) and adjusted *p*-value (Figures S2 and S3). Moreover, the results revealed that some genes were differentially expressed only in the normozoospermic controls, and/or expressed at a low level in the oligoasthenozoospermic men, and vice versa.

### 3.3. Differentially Expressed Proteins in Sperm (Determined Using LC-MS/MS)

**Figure 1:**
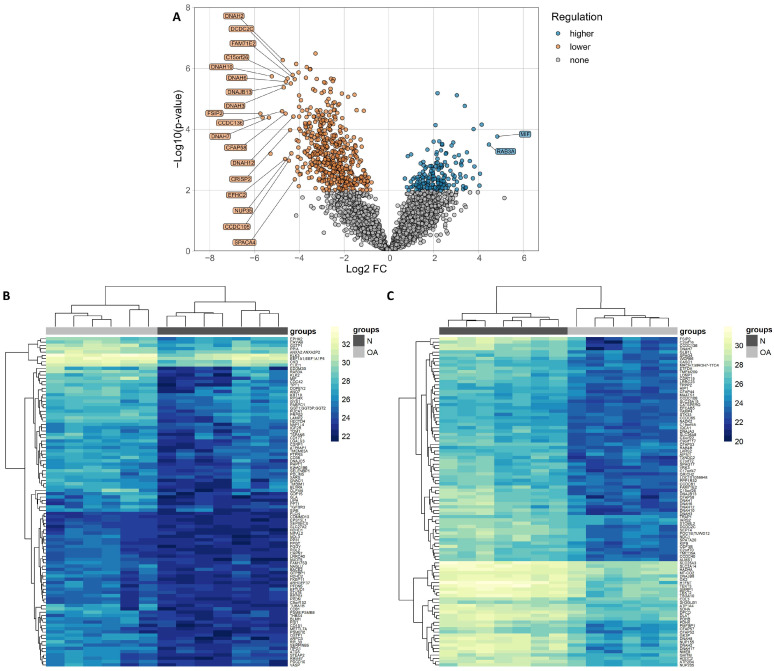
(**A**) Volcano plot shows the differential expression levels of proteins in sperm samples collected from oligoasthenozoospermic men (OA, n = 6) compared to normozoospermic controls (N = 7), as determined by quantitative mass spectrometry. Log2 (fold change) is plotted against the log10 *p*-value. Heatmaps represent hierarchical clustering of the top 100 differentially higher- and lower-expressed proteins (based on adjusted *p*-value) in sperm samples of oligoasthenozoospermic men (OA, n = 6, light gray) compared to normozoospermic controls (N, n = 7, dark gray). Panel (**B**) represents the lower-expressed proteins, and panel (**C**) represents the higher-expressed proteins in sperm samples of oligoasthenozoospermic men compared to normozoospermic controls. The heatmaps were generated based on log2 LFQ intensities, and the rectangle represents the normalized expression level of the protein (the higher the value, the higher the expression level of the protein). Yellow represents higher-expressed proteins, and blue represents lower-expressed proteins.

An independent set of sperm samples was collected from the men with oligoasthenozoospermia (n = 7) and normozoospermic controls (n = 7) to identify proteins with altered expression levels. These samples were analyzed along with another set to study the protein landscape in human sperm (PXD033749) [22]. During LC-MS/MS processing, one sample from the oligoasthenozoospermia group was lost and subsequently excluded from further protein statistical analyses. A total of 3315 proteins, including both significant and non-significant proteins, were detected in the sperm samples of the oligoasthenozoospermic men compared to normozoospermic controls (PXD033749). After applying an unpaired two-tailed *t*-test and adjusting the *p*-value for multiple testing, 692 proteins with significant differences between the oligoasthenozoospermic men and normozoospermic controls were identified (Table S3, adjusted *p* < 0.05). Among these, 512 proteins had significantly lower expression levels, and 180 proteins had significantly higher expression levels (Table S3, adjusted *p* < 0.05). The volcano plot in Figure 1A visualizes the identified lower and higher-expressed proteins. Notably, the non-supervised hierarchical clustering of the top 100 up- or downregulated proteins segregated the oligoasthenozoospermic and normozoospermic sperm samples into separate clusters (Figure 1B,C, respectively). As shown in the heatmap of the hierarchical clustering of differentially expressed lower and higher proteins in Figure 1B,C, the oligoasthenozoospermic men and normozoospermic controls were segregated into two distinct clusters. Similarly, the hierarchical clustering of the 512 proteins with differentially lower expression levels and the 180 proteins with differentially higher expression levels also showed two distinct clusters (Figures S4 and S5).

### 3.4. Significantly Shared Genes and Proteins

**Figure 2:**
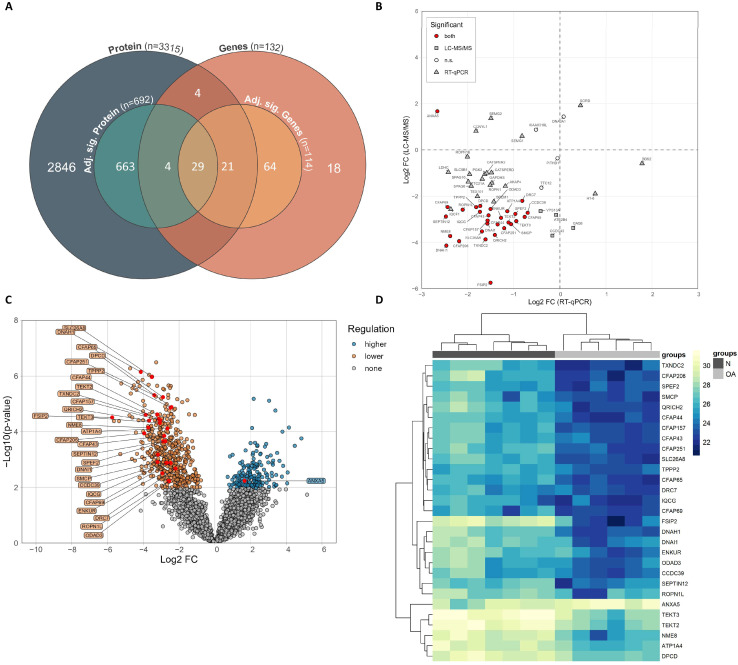
(**A**) Venn diagram showing the identified proteins and genes, including adjusted significant proteins and genes, and the number of overlaps. (**B**) Scatter plot displaying the direction of regulation of the significant shared genes and proteins. (**C**) Volcano plot showing the differential expression levels of proteins in sperm samples collected from oligoasthenozoospermic men (OA, n = 6) compared to normozoospermic men (N, n = 7) as determined by quantitative mass spectrometry. Log2 (fold change) is plotted against the log10 *p*-value. (**D**) Heatmap representing hierarchical clustering of the protein expression of shared and significantly expressed proteins ‘only’ (based on Log2 (fold change) and adjusted *p*-value) in sperm samples of oligoasthenozoospermic men (OA, n = 6) compared to normozoospermic men (N = 6). The heatmaps were generated based on log2 LFQ intensities, and the rectangle represents the normalized expression level of the protein (the higher the value, the higher the expression level of protein). Yellow represents higher-expressed proteins, and blue represents lower-expressed proteins.

Among the 3315 proteins identified in the LC-MS/MS dataset of the sperm from the included subjects, we found 58 proteins that overlapped with the 132 genes associated with sperm motility according to the GO project (Table S4). We next cross-matched the 114 genes with significantly differential expression levels in the oligoasthenozoospermic men compared to those of the normozoospermic controls, as determined by RT-qPCR, and 692 proteins with significantly differential expression levels, as determined by LC-MS/MS (Figure 2A). The cross-matching yielded 29 genes and proteins that were detected by both approaches, as summarized in Table 3. Considering the direction of regulation, there were 28 genes and their corresponding proteins that showed lower expression levels, and one gene and its corresponding protein, namely ANXA5, showed a discordant direction of regulation, i.e., a lower gene expression level and higher protein expression level, as highlighted in Figure 2B,C. The hierarchical clustering of the 29 significantly shared genes and proteins showed two distinguishable clusters, with one cluster containing the oligoasthenozoospermic men and a second cluster containing the normozoospermic controls (Figure 2D).

### 3.5. Correlation of Expression Levels of Significant Genes and Proteins with Basic Semen Parameters

We next analyzed the correlations between the expression levels of the significant shared genes and proteins (listed in Table 3) and the basic semen parameters, including sperm count, motility, and morphology. The Spearman correlation analysis showed weak to strong correlations between the significant shared genes and proteins and the aforementioned semen parameters (Table S5, *p* < 0.05). Specifically, the correlation analysis indicated that the lower the expression level of the genes and proteins, the lower the sperm count, motility, and morphology. With the exception of the ANXA5 protein, the correlation analysis was negatively correlated with the basic semen parameters, whereas ANXA5 was positively correlated with these parameters. Furthermore, by considering only the significant correlations observed in both genes and proteins with sperm motility, 19 genes and proteins were positively correlated with sperm motility, as summarized in Table 4.

### 3.6. Enrichment Analysis of the Significantly Shared Genes and Proteins

**Figure 3:**
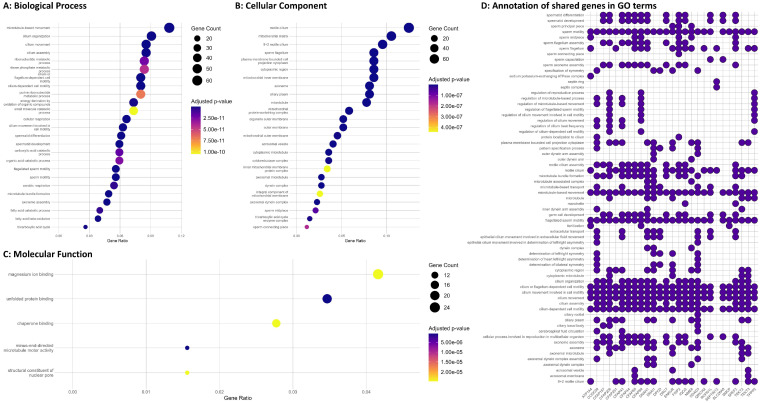
Gene Ontology (GO) overrepresentation analysis was conducted on the significantly adjusted proteins. The results show (**A**) the top 25 enriched terms in Biological Process, (**B**) the top 25 enriched terms in Cellular Component, and (**C**) the top 5 enriched terms in Molecular Function. The gene ratio on the *x*-axis was calculated by dividing the number of matched proteins in each term by the total number of annotated input genes in that section. The dot size represents the number of proteins found in each term, and the color indicates the adjusted *p*-values. Additionally, (**D**) a heatmap plot was created to display the functional classification of shared genes and proteins involved in sperm motility, with selected terms from the GO project.

To further characterize the regulated proteins identified by LC-MS/MS, the overrepresentation of the Gene Ontology (GO) annotation terms among the 692 proteins relative to all the identified proteins was calculated (Table S6). In the category of biological processes (BP), the proteins were functionally related to sperm and sperm-related biological processes, including cilium- or flagellum-dependent cell motility, flagellated sperm motility, sperm motility, spermatid differentiation, and development. Moreover, several proteins involved in lipid metabolism relevant to spermatogenesis and sperm function were identified, as shown in Figure 3A. Of these lipid metabolisms, relevant processes which provide the main source of ATP for male gametes were observed, including the fatty acid catabolic process, fatty acid oxidation, fatty acid metabolic process, fatty acid beta-oxidation, Acyl-CoA dehydrogenase, and fatty acid derivative metabolic process (Table S6). Many other proteins were also involved in energetic metabolism, as shown in Table S6. As for the cellular components (CC), the significantly expressed proteins were functionally related to the motile cilium, sperm flagellum, axoneme, mitochondrial matrix, mitochondrial inner membrane, axonemal microtubule, axonemal dynein complex, sperm head, sperm midpiece, sperm connecting piece, and many other cellular components affecting sperm parts (Figure 3B and Table S6). Additionally, the differentially expressed proteins were found to be associated with the structural constituent of the nuclear pore, magnesium ion binding, unfolded protein binding, minus-end-directed microtubule motor activity, and many other processes related to energetic metabolism and binding (Figure 3C and Table S6). Finally, the visualization of the GO terms linked to the significant shared genes and proteins found to be significantly deregulated in the RT-qPCR and LC-MS/MS study revealed many terms related to sperm-related biological processes (Figure 3D).

**Figure 4:**
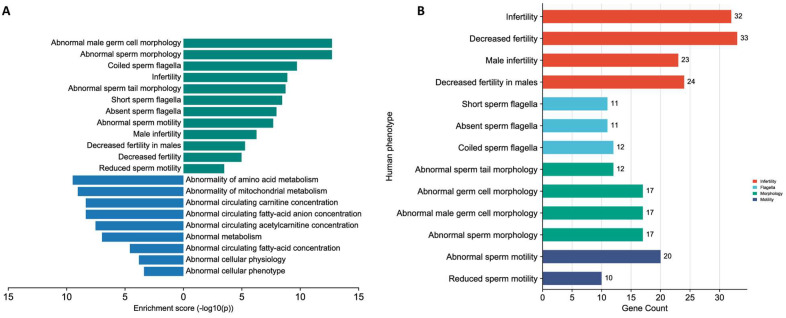
(**A**) Human phenotypes associated with sperm dysfunction and energy metabolism; (**B**) Gene count associated with sperm motility and flagella, morphology dysfunction, and male infertility phenotypes.

The ToppGene Suite was used to classify the differentially and significantly identified proteins (i.e., 692 proteins determined by the LC-MS/MS analysis) and to identify their associations with distinct sperm pathological phenotypes in humans. The results showed that many of the identified proteins were associated with sperm motility and flagella, morphology dysfunction, metabolism, and male infertility phenotypes, as presented in Figure 4A,B.

## 4. Discussion

Spermatogenic defects in males with decreased fertility are caused by a series of genetic and epigenetic alterations [23]. To contribute to a more comprehensive genetic characterization, we used high-throughput techniques to validate a set of genes associated with sperm motility in sperm samples collected from men with oligoasthenozoospermia and normozoospermia. A total of 58 shared genes and proteins (i.e., significant and non-significant genes and proteins) were identified when we cross-matched the 3315 proteins that were detected in the sperm samples of the oligoasthenozoospermic men, compared to the normozoospermic controls (listed in PXD033749), with a total of 132 genes annotated in Homo sapiens sperm motility (GO:0097722) and flagellated sperm motility (GO:0030317) (Table S4). The cross-match between the 114 genes that showed a significantly differential expression level in the oligoasthenozoospermic men compared to the normozoospermic controls as determined by RT-qPCR, and the 692 proteins with significantly differential expression levels as determined by LC-MS/MS yielded 29 genes and proteins that were detected by both approaches (Table 3). As anticipated, most of the significant shared genes and proteins that were dysregulated in the sperm samples are required for sperm flagellum and motility and/or play important roles in energetic metabolism, since ATP is evidently needed to support sperm motility. Indeed, most of the Gene Ontology (GO) terms detected by the analysis were related to sperm and sperm-related biological processes (Table S6). Some genes with lower expression levels, namely DPCD, NME8, and ENKUR, had not yet been clearly related to biological functions in sperm production, function, and/or infertility-related manifestations. Of note, Enkurin (ENKUR) had been reported to play a role in the Ca_2_^+^-sensitive signal transduction machinery in sperm [23]. Other genes had already been related to sperm production, function, and/or infertility-related manifestations in humans or other mammals. Additionally, we identified enriched human phenotypes related to spermatogenesis and sperm-related abnormalities, including abnormal sperm motility and morphology (Figure 4 and Table S6).

Using GO enrichment analyses of significantly expressed proteins, the sperm motility (GO:0097722) and flagellated sperm motility (GO:0030317), along with other enriched categories associated with spermatogenesis and germ cells at different spermatogenic stages, were identified (Figure 3). Since the flagellum in sperm is responsible for motility, alterations in the genes necessary for sperm flagella function may lead to reduced sperm motility or even the immobility of sperm. Out of these genes, A-kinase anchor protein 4 (AKAP4) is one of the major structural components of the sperm fibrous sheath [24] and its normal expression level is crucial for maintaining the expression level of glutamine-rich 2 (QRICH2) [25]. Reduced AKAP4 and QRICH2 protein levels cause dysplasia of the fibrous sheath, which ultimately leads to decreased sperm motility and male infertility [25]. In men with oligozoospermia, the expression level of AKAP4 was 10-fold lower than that in normozoospermic controls [26]. 

Numerous genes identified in our study are associated with multiple morphological abnormalities of the sperm flagella (MMAF) syndrome, including AKAP4, QRICH2, and other genes, namely the cilia- and flagella-associated protein 43 (CFAP43), the cilia- and flagella-associated protein 44 (CFAP44), the cilia- and flagella-associated protein 69 (CFAP69), the coiled-coil domain containing 39 (CCDC39), and the fibrous sheath-interacting protein 2 (FSIP2) [27,28,29,30,31]. MMAF is associated with male infertility and demonstrates a variety of flagella morphological abnormalities, including absent, short, bent, coiled, and irregular flagella [32]. Moreover, in patients with severe sperm motility disorders, pathogenic variants were observed in CFAP43, CFAP44, QRICH2, CCDC39, DNAH1, DNAI1, and FSIP2 genes [2,33,34,35]. Shen et al., reported that bi-allelic mutations in cilia- and flagella-associated protein 206 (CFAP206) led to impaired fertility by inducing morphological and functional defects in the sperm flagellum [36]. Zhang et al., suggested that CFAP65 is involved in sperm flagellum structure and assembly and its loss-of-function mutations cause male infertility associated with the MMAF phenotype [37]. Moreover, CFAP65 plays a crucial role during sperm head shaping, acrosome formation, and flagella assembly. CFAP65 interacts with the mitochondrial protein TPPP2 (tubulin polymerization-promoting protein family member 2) and forms a functional cytoplasmic protein network/complex [38]. FSIP2 encodes a protein that has been associated with the formation of the sperm fibrous sheath and is expressed in the cytoplasm of primary germ cells and the flagella of spermatids during the spermatogenesis process [39,40]. In clinical practice, specifically, in couples attending intracytoplasmic sperm injection (ICSI), pregnancy was not achieved after embryo transfer when sperm was collected from patients with heterozygous mutations in FSIP2 [39]. Previous studies have reported that sperm flagellar 2 (SPEF2) plays a key role in the development of the sperm tail and cilia and that alterations in its expression level result in severe asthenoteratozoospermia [41,42,43]. Mutations in SPEF2 have been reported to cause MMAF with or without primary ciliary dyskinesia (PCD) symptoms [41,42,43], indicating that its loss-of-function mutations lead to spermatogenic dysfunction and male infertility. Kherraf et al., and Auguste et al., found that deleterious variants in CFAP251/WDR66 are associated with MMAF [44,45], and CFAP251/WDR66 was completely absent from the sperm collected from patients with asthenozoospermia [36]. Our findings are in agreement with those of previous studies reporting an association between the lower expression level of the aforementioned cilia- and flagella-associated proteins and men with reduced fertility.

Our finding of tektin 2 (TEKT2) and tektin 3 (TEKT3) among the differentially expressed deregulated genes and proteins in the oligoasthenozoospermic men compared to normozoospermic controls is consistent with the findings of previous studies, which indicated a possible involvement of genes in the development and regulation of the axoneme, i.e., a microtubule-based cytoskeletal structure that forms the core of a flagellum. The cytoskeletal proteins TEKT2 and TEKT3 are testis-specific and play a role in the flagellar formation and/or ciliary movement during the process of spermatogenesis [46,47]. Mutations in the flagellar tektin proteins seem to be associated with asthenozoospermia and male subfertility [48]. In agreement with our finding, lower expression levels of cytoskeletal tektin proteins were observed in the oligoasthenozoospermic men compared to those of the normozoospermic controls. Two more genes are important for the structural integrity of the central apparatus in the sperm tail and flagellar motility function, namely sperm-associated antigen 6 (SPAG6) and sperm-associated antigen 16 (SPAG16). SPAG6 was detected in the spermatogonia, spermatocytes, and sperm flagella, whereas SPAG16 was found in the spermatocytes and sperm flagella [49,50]. Xu et al., reported a lower expression of SPAG6 in spermatozoa from men harboring SPAG6 variants and suggested that SPAG6 variants are a potential pathogenic factor for syndromic severe asthenozoospermia and the non-syndromic asthenoteratozoospermia-associated MMAF phenotype [50].

Genetic studies have suggested deficiencies in several genes, particularly genes associated with energy metabolism, energy production, and ion channels, as major factors affecting sperm motility and leading to asthenozoospermia. Our findings also support the idea that many of the genes associated with motility defects in oligoasthenozoospermic men play a role in metabolism, energy production, and ion channels. Of these genes, lactate dehydrogenase C (LDHC) is a glycolytic enzyme (protein) that catalyzes the reduction of pyruvate to lactate with the concurrent oxidation of NADH to NAD+. LDHC localizes throughout the flagellum presumably to produce energy that is needed for normal sperm function [51]. LDHC along with other glycolytic enzymes, namely glyceraldehyde-3-phosphate dehydrogenase, testis-specific (GAPDHS) and phosphoglycerate kinase 2 (PGK2), are associated with sperm quality and function and are exclusively expressed in the post-meiotic germ cells of human testes [52]. In human sperm, LDHC, GAPDHS, and PGK2 are localized in the longest portion of the sperm tail where the energy is produced for sperm motility by glycolysis [52]. Liu et al., found lower expression levels of LDHC, GAPDHS, and PGK2 in the human testes and sperm with poor motility in men with asthenozoospermia, suggesting that alteration in the expression levels of LDHC, GAPDHS, and PGK2 may lead to male fertility impairment by disrupting the sperms’ ability to move [52,53]. Our findings are consistent with those of previous studies in showing lower expression levels of LDHC, GAPDHS, and PGK2 in oligoasthenozoospermic men compared to normozoospermic controls. Moreover, solute carrier family 26-member 8 (SLC26A8) is related to anion fluxes and glycolytic enzymes [54] and is highly expressed in male germ cells from the early stages of spermatogenesis [55]. Gao et al., reported a lower expression level of SLC26A8 in spermatozoa obtained from patients harboring biallelic SLC26A8 mutations [56], which may manifest as a recessive genetic cause of severe asthenozoospermia. The serine protease 55 (PRSS55) is specifically expressed in testicular spermatids and epididymal spermatozoa and plays an important role in the structural differentiation and energy metabolism of sperm [57]. The deficiency and/or deletion of PRSS55 leads to male fertility impairment associated with increased sperm malformation and decreased sperm motility [57]. The testis-specific post-meiotic marker spermatid maturation 1 (SPEM1) showed a lower expression level in the testicular tissue of azoospermic men with non-obstructive azoospermia (NOA) as compared to those with obstructive azoospermia (OA) [58]. Additionally, the testis-expressed protein 101 (TEX101) has been validated as a biomarker for the differential diagnosis of NOA versus OA [59,60]. The rheophilic-associated tail protein 1-like (ROPN1L) and the orthologous rheophilic-associated tail protein 1 (ROPN1) are located in the fibrous sheath of sperm. ROPN1 along with other genes regulates sperm motility via the cAMP/PKA signal pathway, indicating that protein phosphorylation may be an important mechanism underlying sperm diversity [61]. The sperm mitochondria-associated cysteine-rich protein (SMCP) along with AKAP4 and FSIP2 plays a vital role in sperm development and motility of the flagellum [61]. Additionally, ROPN1L knockout mice displayed mildly impaired sperm motility, while ROPN1L and ROPN1 knockout mice had immotile spermatozoa [62]. Septin 12 (SEPT12) is a testis-specific GTP-binding protein that is required to maintain the structural integrity of sperm during spermatogenesis, more specifically, in the morphogenesis of sperm heads and the elongation of sperm tails [63,64]. Loss-of-function mutations of SEPT12 disrupted sperm structural integrity and led to poor male fertility [64]. Moreover, mutations in SEPT12 are associated with acrosome defects [65] and are more frequent in patients with spermatogenic impairment [66]. The expression level of *SEPT12* transcripts was significantly lower in the testicular tissues of infertile men compared to fertile men and the loss of SEPT12 in sperm was observed in men with asthenozoospermia [67]. 

Sperm flagella movement is launched by mounting proteins and ions in an appropriate concentration [68]. Among them, ATPase Na^+^/K^+^ and ATPase Ca_2_^+^ are enzymes that are crucial in maintaining the plasma membrane gradient and conducting sperm motility, such as activation and hyperactivation. In our study, a significant decrease in ATPase Na^+^/K^+^ transporting subunit alpha 4 (ATP1A4) was observed in oligoasthenozoospermic men, suggesting poor activities of Na^+^/K^+^-ATPase and dynein ATPase. ATP1A4 is the catalytic subunit of the Na^+^/K^+^-ATPase membrane protein, which plays a crucial role in the regulation of the Na^+^ and K^+^ ion exchange across the plasma membrane in an ATP-dependent reaction [69]. This regulation process is highly needed to maintain sperm function, especially sperm motility [70]. In men with seminoma, a significant decrease in ATP1A4 in sperm may explain the reduction in sperm motility [71]. Similarly, ATP1A4 was significantly decreased in asthenozoospermic testicular cancer patients [72]. A sperm-specific calcium channel, the cation channel sperm-associated auxiliary subunit delta (CATSPERD) along with other CatSper genes regulates calcium influx into sperm and plays a functional role in the regulation of sperm motility, hyperactivity, and male fertility [73]. Deletion or loss-of-function mutations in genes for the CatSper channels cause infertility due to the lack of sperm hyperactivation [73,74,75]. Additionally, IQ motif containing G (IQCG) is essential for sperm flagellum formation and important for sperm nucleus and acrosome elongation through participation in sperm calcium signaling [76]. Another sperm-specific oxidoreductase gene, namely thioredoxin domain-containing 2 (TXNDC2), was detected only in the tail of elongating spermatids and spermatozoa [77]. TXNDC2, along with protamine 1 and 2 (PRM1 and PRM2), was significantly decreased in patients with different patterns of NOA compared to OA patients, suggesting that TXNDC2, PRM1, and PRM2 have a robust power to predict sperm retrieval and are correlated with phenotypes of severe azoospermia [78].

## 5. Conclusions

In conclusion, we have used a combination of RT-qPCR and LC-MS/MS analyses to validate the genes and proteins associated with sperm motility and flagellum in oligoasthenozoospermic and normozoospermic men. Our findings have revealed a group of significant shared genes and proteins with similarly altered expression levels. The genes and/or proteins that showed lower expression levels in oligoasthenozoospermic men are involved in maintaining the sperm integrity and cytoskeleton, energy-related metabolism, plasma membrane gradient, and cation channel. These results strongly suggest that these genes and/or proteins play a crucial role in the molecular and biological function of sperm, and that any alteration in their expression levels can lead to spermatogenic dysfunction, reduced sperm motility, failure of capacitation, and fertilization. In the future, these genes and/or proteins could serve as potential diagnostic and prognostic biomarkers and perhaps lead to the identification of novel therapeutic targets.

## Figures and Tables

**Table 1:** Semen characteristics of included subjects.

Characteristic	Normozoospermic Men (Healthy Controls, n = 99)	Oligoasthenozoospermic Men (Subfertile Men, n = 99)	*p*-Value
Age (Year)	35.49 ± 6.31	34.70 ± 6.73	3.91 × 10^−1^
Volume (mL)	3.45 ± 1.47	3.13 ± 1.34	1.16 × 10^−1^
pH	8.71 ± 0.39	8.71 ± 0.38	9.70 × 10^−1^
Count (10^6^/mL)	66.51 ± 29.87	9.26 ± 3.56	3.79 × 10^−46^
Motility (%, motile)	56.57 ± 10.50	20.53 ± 4.81	1.26 × 10^−77^
Morphology (%, normal)	8.08 ± 4.62	5.86 ± 2.26	3.63 × 10^−5^

An unpaired two-tailed *t*-test was used to calculate the *p*-value. Data were presented as mean ± standard deviation. *p* < 0.05 was considered statistically significant.

**Table 2:** The genes with significant expression in sperm samples obtained from men with oligoasthenozoospermia (n = 92) were compared to those of normozoospermic men (n = 92), as determined by RT-qPCR.

Entrez Gene ID	Gene Symbol and Name	Log2 (Fold Change)	*p*-Value	Adjusted *p*-Value	Regulation
100	ADA (adenosine deaminase)	−1.24	4.42 × 10^−4^	6.63 × 10^−4^	Lower
8852	AKAP4 (A-kinase anchoring protein 4)	−1.18	7.05 × 10^−4^	1.01 × 10^−3^	Lower
308	ANXA5 (annexin A5)	−2.66	2.22 × 10^−10^	2.25 × 10^−9^	Lower
84071	ARMC2 (armadillo repeat containing 2)	−1.40	6.31 × 10^−6^	1.41 × 10^−5^	Lower
55870	ASH1L (ASH1 like histone lysine methyltransferase)	−2.06	2.39 × 10^−10^	2.25 × 10^−9^	Lower
480	ATP1A4 (ATPase Na+/K+ transporting subunit alpha 4)	−1.14	7.99 × 10^−4^	1.10 × 10^−3^	Lower
8911	CACNA1I (calcium voltage-gated channel subunit alpha1 I)	−1.11	2.07 × 10^−2^	2.51 × 10^−2^	Lower
117144	CATSPER1 (cation channel sperm associated 1)	−2.01	1.34 × 10^−7^	4.48 × 10^−7^	Lower
117155	CATSPER2 (cation channel sperm associated 2)	−1.59	2.47 × 10^−5^	4.66 × 10^−5^	Lower
347732	CATSPER3 (cation channel sperm associated 3)	−1.67	2.70 × 10^−7^	8.09 × 10^−7^	Lower
378807	CATSPER4 (cation channel sperm associated 4)	−1.73	1.87 × 10^−4^	2.98 × 10^−4^	Lower
257062	CATSPERD (cation channel sperm associated auxiliary subunit delta)	−1.48	2.80 × 10^−5^	5.18 × 10^−5^	Lower
25858	CATSPERZ (catsper channel auxiliary subunit zeta)	−2.04	4.33 × 10^−10^	3.57 × 10^−9^	Lower
339829	CCDC39 (coiled-coil domain containing 39)	−0.70	3.36 × 10^−2^	3.96 × 10^−2^	Lower
151195	CCNYL1 (cyclin Y like 1)	−1.82	1.48 × 10^−10^	1.63 × 10^−9^	Lower
22994	CEP131 (centrosomal protein 131)	−1.33	1.86 × 10^−7^	5.85 × 10^−7^	Lower
286207	CFAP157 (cilia and flagella associated protein 157)	−1.57	2.77 × 10^−4^	4.30 × 10^−4^	Lower
154313	CFAP206 (cilia and flagella associated protein 206)	−2.19	7.53 × 10^−10^	5.85 × 10^−9^	Lower
80217	CFAP43 (cilia and flagella associated protein 43)	−1.55	5.90 × 10^−7^	1.59 × 10^−6^	Lower
55779	CFAP44 (cilia and flagella associated protein 44)	−1.35	4.11 × 10^−6^	9.86 × 10^−6^	Lower
255101	CFAP65 (cilia and flagella associated protein 65)	−0.76	2.72 × 10^−2^	3.24 × 10^−2^	Lower
79846	CFAP69 (cilia and flagella associated protein 69)	−2.44	4.88 × 10^−8^	1.84 × 10^−7^	Lower
1139	CHRNA7 (cholinergic receptor nicotinic alpha 7 subunit)	−1.78	5.20 × 10^−9^	2.86 × 10^−8^	Lower
54514	DDX4 (DEAD-box helicase 4)	−1.24	6.35 × 10^−4^	9.21 × 10^−4^	Lower
25981	DNAH1 (dynein axonemal heavy chain 1)	−2.46	1.35 × 10^−9^	8.85 × 10^−9^	Lower
8701	DNAH11 (dynein axonemal heavy chain 11)	−1.24	2.77 × 10^−3^	3.53 × 10^−3^	Lower
1767	DNAH5 (dynein axonemal heavy chain 5)	−1.77	1.75 × 10^−7^	5.64 × 10^−7^	Lower
27019	DNAI1 (dynein axonemal intermediate chain 1)	−1.57	1.08 × 10^−5^	2.30 × 10^−5^	Lower
25911	DPCD (deleted in primary ciliary dyskinesia homolog (mouse))	−1.73	2.37 × 10^−5^	4.60 × 10^−5^	Lower
84229	DRC7 (dynein regulatory complex subunit 7)	−0.82	1.37 × 10^−3^	1.87 × 10^−3^	Lower
285588	EFCAB9 (EF-hand calcium binding domain 9)	−2.30	2.10 × 10^−5^	4.15 × 10^−5^	Lower
219670	ENKUR (enkurin, TRPC channel interacting protein)	−1.28	1.86 × 10^−8^	7.45 × 10^−8^	Lower
387712	ENO4 (enolase 4)	−1.80	1.00 × 10^−6^	2.59 × 10^−6^	Lower
57119	EPPIN (epididymal peptidase inhibitor)	−1.25	4.67 × 10^−13^	2.05 × 10^−11^	Lower
401024	FSIP2 (fibrous sheath interacting protein 2)	−1.50	2.70 × 10^−5^	5.02 × 10^−5^	Lower
26330	GAPDHS (glyceraldehyde-3-phosphate dehydrogenase, spermatogenic)	−1.48	8.77 × 10^−7^	2.32 × 10^−6^	Lower
8100	IFT88 (intraflagellar transport 88)	−1.92	7.51 × 10^−9^	3.54 × 10^−8^	Lower
132141	IQCF1 (IQ motif containing F1)	−2.37	2.93 × 10^−7^	8.60 × 10^−7^	Lower
84223	IQCG (IQ motif containing G)	−1.73	2.58 × 10^−8^	1.00 × 10^−7^	Lower
3948	LDHC (lactate dehydrogenase C)	−2.42	3.66 × 10^−10^	3.22 × 10^−9^	Lower
23639	LRRC6/DNAAF11 (dynein axonemal assembly factor 11)	−1.70	1.36 × 10^−7^	4.48 × 10^−7^	Lower
644890	MEIG1 (meiosis/spermiogenesis associated 1)	−1.60	1.11 × 10^−7^	3.93 × 10^−7^	Lower
4233	MET (MET proto-oncogene, receptor tyrosine kinase)	−1.64	3.02 × 10^−7^	8.68 × 10^−7^	Lower
51314	NME8 (NME/NM23 family member 8)	−2.38	5.98 × 10^−6^	1.36 × 10^−5^	Lower
79730	NSUN7 (NOP2/Sun RNA methyltransferase family member 7)	−0.77	1.50 × 10^−4^	2.44 × 10^−4^	Lower
115948	ODAD3/CCDC151 (outer dynein arm docking complex subunit 3)	−1.51	3.65 × 10^−2^	4.23 × 10^−2^	Lower
441531	PGAM4 (phosphoglycerate mutase family member 4)	−1.25	1.45 × 10^−3^	1.96 × 10^−3^	Lower
5232	PGK2 (phosphoglycerate kinase 2)	−1.63	4.26 × 10^−3^	5.35 × 10^−3^	Lower
139212	PIH1D3/DNAAF6 (dynein axonemal assembly factor 6)	−1.13	1.54 × 10^−2^	1.89 × 10^−2^	Lower
50487	PLA2G3 (phospholipase A2 group III)	−2.81	5.11 × 10^−9^	2.86 × 10^−8^	Lower
63978	PRDM14 (PR/SET domain 14)	−1.07	1.07 × 10^−2^	1.32 × 10^−2^	Lower
58531	PRM3 (protamine 3)	−2.13	1.67 × 10^−11^	2.46 × 10^−10^	Lower
203074	PRSS55 (serine protease 55)	−1.21	2.46 × 10^−3^	3.19 × 10^−3^	Lower
84074	QRICH2 (glutamine rich 2)	−1.41	1.52 × 10^−5^	3.09 × 10^−5^	Lower
54763	ROPN1 (rhophilin associated tail protein 1)	−1.51	1.56 × 10^−8^	6.43 × 10^−8^	Lower
152015	ROPN1B (rhophilin associated tail protein 1B)	−2.01	1.69 × 10^−9^	1.01 × 10^−8^	Lower
83853	ROPN1L (rhophilin associated tail protein 1 like)	−2.11	1.41 × 10^−8^	6.00 × 10^−8^	Lower
6406	SEMG1 (semenogelin 1)	−0.82	2.03 × 10^−4^	3.20 × 10^−4^	Lower
6407	SEMG2 (semenogelin 2)	−1.50	2.86 × 10^−5^	5.18 × 10^−5^	Lower
124404	SEPTIN12 (septin 12)	−2.47	1.17 × 10^−11^	2.20 × 10^−10^	Lower
9389	SLC22A14 (solute carrier family 22 member 14)	−1.44	6.77 × 10^−5^	1.21 × 10^−4^	Lower
116369	SLC26A8 (solute carrier family 26 member 8)	−1.69	1.13 × 10^−7^	3.93 × 10^−7^	Lower
150159	SLC9B1 (solute carrier family 9 member B1)	−1.96	1.71 × 10^−12^	5.65 × 10^−11^	Lower
285335	SLC9C1 (solute carrier family 9 member C1)	−1.26	4.19 × 10^−4^	6.37 × 10^−4^	Lower
81892	SLIRP (SRA stem-loop interacting RNA binding protein)	−2.12	1.61 × 10^−6^	4.09 × 10^−6^	Lower
4184	SMCP (sperm mitochondria associated cysteine rich protein)	−1.06	9.29 × 10^−4^	1.31 × 10^−3^	Lower
79582	SPAG16 (sperm associated antigen 16)	−1.99	8.52 × 10^−9^	3.75 × 10^−8^	Lower
9576	SPAG6 (sperm associated antigen 6)	−1.92	6.59 × 10^−9^	3.34 × 10^−8^	Lower
79925	SPEF2 (sperm flagellar 2)	−0.97	4.20 × 10^−4^	6.37 × 10^−4^	Lower
374768	SPEM1 (spermatid maturation 1)	−1.43	4.40 × 10^−6^	1.04 × 10^−5^	Lower
6863	TAC1 (tachykinin precursor 1)	−2.25	2.63 × 10^−6^	6.43 × 10^−6^	Lower
6866	TAC3 (tachykinin precursor 3)	−2.27	5.52 × 10^−6^	1.28 × 10^−5^	Lower
255061	TAC4 (tachykinin precursor 4)	−1.09	7.27 × 10^−6^	1.57 × 10^−5^	Lower
6869	TACR1 (tachykinin receptor 1)	−1.17	8.44 × 10^−3^	1.05 × 10^−2^	Lower
6865	TACR2 (tachykinin receptor 2)	−1.73	6.62 × 10^−6^	1.46 × 10^−5^	Lower
6870	TACR3 (tachykinin receptor 3)	−0.68	3.57 × 10^−2^	4.18 × 10^−2^	Lower
54457	TAF7L (TATA-box binding protein associated factor 7 like)	−1.97	2.27 × 10^−7^	6.97 × 10^−7^	Lower
202500	TCTE1 (t-complex-associated-testis-expressed 1)	−1.27	1.60 × 10^−4^	2.58 × 10^−4^	Lower
27285	TEKT2 (tektin 2)	−1.11	7.57 × 10^−5^	1.33 × 10^−4^	Lower
64518	TEKT3 (tektin 3)	−0.95	2.78 × 10^−3^	3.53 × 10^−3^	Lower
83639	TEX101 (testis expressed 101)	−1.79	1.72 × 10^−5^	3.43 × 10^−5^	Lower
283471	TMPRSS12 (transmembrane serine protease 12)	−1.39	9.83 × 10^−5^	1.71 × 10^−4^	Lower
7141	TNP1 (transition protein 1)	−2.16	4.55 × 10^−12^	1.20 × 10^−10^	Lower
7142	TNP2 (transition protein 2)	−0.97	1.80 × 10^−3^	2.40 × 10^−3^	Lower
122664	TPPP2 (tubulin polymerization promoting protein family member 2)	−1.82	8.67 × 10^−10^	6.36 × 10^−9^	Lower
283629	TSSK4 (testis specific serine kinase 4)	−1.80	4.93 × 10^−7^	1.36 × 10^−6^	Lower
199223	TTC21A (tetratricopeptide repeat domain 21A)	−1.69	9.47 × 10^−10^	6.58 × 10^−9^	Lower
25809	TTLL1 (TTL family tubulin polyglutamylase complex subunit L1)	−2.21	1.55 × 10^−11^	2.46 × 10^−10^	Lower
23093	TTLL5 (tubulin tyrosine ligase like 5)	−1.58	7.78 × 10^−9^	3.54 × 10^−8^	Lower
164395	TTLL9 (tubulin tyrosine ligase like 9)	−1.07	6.04 × 10^−4^	8.86 × 10^−4^	Lower
84203	TXNDC2 (thioredoxin domain containing 2)	−1.61	3.34 × 10^−7^	9.38 × 10^−7^	Lower
7320	UBE2B (ubiquitin conjugating enzyme E2 B)	−2.12	7.55 × 10^−9^	3.54 × 10^−8^	Lower
144406	WDR66/CFAP251 (cilia and flagella associated protein 251)	−1.21	1.26 × 10^−4^	2.09 × 10^−4^	Lower
7490	WT1 (WT1 transcription factor)	−1.33	1.27 × 10^−4^	2.09 × 10^−4^	Lower
109	ADCY3 (adenylate cyclase 3)	1.60	1.28 × 10^−3^	1.76 × 10^−3^	Higher
338	APOB (apolipoprotein B)	1.39	1.91 × 10^−3^	2.53 × 10^−3^	Higher
583	BBS2 (Bardet-Biedl syndrome 2)	1.78	5.21 × 10^−8^	1.91 × 10^−7^	Higher
1235	CCR6 (C-C motif chemokine receptor 6)	1.23	1.24 × 10^−5^	2.58 × 10^−5^	Higher
1672	DEFB1 (defensin beta 1)	1.30	1.25 × 10^−5^	2.58 × 10^−5^	Higher
3010	H1-6 (H1.6 linker histone, cluster member)	0.77	9.96 × 10^−4^	1.38 × 10^−3^	Higher
3622	ING2 (inhibitor of growth family member 2)	0.54	1.08 × 10^−4^	1.85 × 10^−4^	Higher
11172	INSL6 (insulin like 6)	0.58	2.30 × 10^−2^	2.76 × 10^−2^	Higher
55726	INTS13 (integrator complex subunit 13)	2.30	2.08 × 10^−11^	2.50 × 10^−10^	Higher
54742	LY6K (lymphocyte antigen 6 family member K)	1.57	6.44 × 10^−9^	3.34 × 10^−8^	Higher
9148	NEURL1 (neuralized E3 ubiquitin protein ligase 1)	0.71	1.95 × 10^−3^	2.55 × 10^−3^	Higher
261734	NPHP4 (nephrocystin 4)	1.49	1.95 × 10^−6^	4.86 × 10^−6^	Higher
11158	RABL2B (RAB, member of RAS oncogene family like 2B)	2.46	1.96 × 10^−11^	2.50 × 10^−10^	Higher
9104	RGN (regucalcin)	1.58	1.41 × 10^−9^	8.85 × 10^−9^	Higher
338879	RNASE10 (ribonuclease A family member 10 (inactive))	2.65	8.57 × 10^−14^	5.66 × 10^−12^	Higher
390443	RNASE9 (ribonuclease A family member 9 (inactive))	1.20	2.44 × 10^−5^	4.66 × 10^−5^	Higher
85413	SLC22A16 (solute carrier family 22 member 16)	3.18	7.59 × 10^−12^	1.67 × 10^−10^	Higher
133308	SLC9B2 (solute carrier family 9 member B2)	3.25	3.67 × 10^−18^	4.85 × 10^−16^	Higher
6652	SORD (sorbitol dehydrogenase)	0.45	5.32 × 10^−4^	7.89 × 10^−4^	Higher
91978	TPGS1 (tubulin polyglutamylase complex subunit 1)	0.99	1.25 × 10^−4^	2.09 × 10^−4^	Higher

An unpaired two-tailed *t*-test was used to calculate the *p*-value. A false discovery rate (FDR) correction was used to adjust the *p*-values. An adjusted *p*-value < 0.05 was considered statistically significant.

**Table 3:** The significant shared genes and proteins in sperm samples of men with oligoasthenozoospermia compared to those of normozoospermic men were determined by RT-qPCR and LC-MS/MS.

Entrez ID	Gene Symbol	Genes (RT-qPCR)				Proteins (LC-MS/MS)			
		Log2 (Fold Change)	*p*-Value	Adjusted *p*-Value	Regulation	Log2 (Fold Change)	*p*-Value	Adjusted *p*-Value	Regulation
308	ANXA5	−2.66	2.22 × 10^−10^	2.25 × 10^−9^	Lower	1.67	5.79 × 10^−3^	3.39 × 10^−2^	Higher
480	ATP1A4	−1.14	7.99 × 10^−4^	1.13 × 10^−3^	Lower	−2.65	7.63 × 10^−5^	2.51 × 10^−3^	Lower
339829	CCDC39	−0.70	3.36 × 10^−2^	3.96 × 10^−2^	Lower	−2.72	1.33 × 10^−3^	1.31 × 10^−2^	Lower
286207	CFAP157	−1.57	2.77 × 10^−4^	4.30 × 10^−4^	Lower	−3.05	3.88 × 10^−5^	1.81 × 10^−3^	Lower
154313	CFAP206	−2.19	7.53 × 10^−10^	5.85 × 10^−9^	Lower	−3.95	1.14 × 10^−4^	3.14 × 10^−3^	Lower
144406	CFAP251	−1.21	1.26 × 10^−4^	2.09 × 10^−4^	Lower	−3.38	4.85 × 10^−6^	6.99 × 10^−4^	Lower
80217	CFAP43	−1.55	5.90 × 10^−7^	1.59 × 10^−6^	Lower	−2.83	1.41 × 10^−4^	3.62 × 10^−3^	Lower
55779	CFAP44	−1.35	4.11 × 10^−6^	9.86 × 10^−6^	Lower	−3.22	2.40 × 10^−5^	1.55 × 10^−3^	Lower
255101	CFAP65	−0.76	2.72 × 10^−2^	3.24 × 10^−2^	Lower	−2.90	5.73 × 10^−6^	7.60 × 10^−4^	Lower
79846	CFAP69	−2.44	4.88 × 10^−8^	1.84 × 10^−7^	Lower	−2.47	1.33 × 10^−3^	1.31 × 10^−2^	Lower
25981	DNAH1	−2.46	1.35 × 10^−9^	8.85 × 10^−9^	Lower	−4.14	7.19 × 10^−7^	5.05 × 10^−4^	Lower
27019	DNAI1	−1.57	1.08 × 10^−5^	2.30 × 10^−5^	Lower	−3.18	6.63 × 10^−4^	8.63 × 10^−3^	Lower
25911	DPCD	−1.73	2.37 × 10^−5^	4.60 × 10^−5^	Lower	−2.42	1.30 × 10^−5^	1.16 × 10^−3^	Lower
84229	DRC7	−0.82	1.37 × 10^−3^	1.87 × 10^−3^	Lower	−2.20	2.11 × 10^−3^	1.76 × 10^−2^	Lower
219670	ENKUR	−1.28	1.86 × 10^−8^	7.45 × 10^−8^	Lower	−2.94	3.09 × 10^−3^	2.28 × 10^−2^	Lower
401024	FSIP2	−1.50	2.70 × 10^−5^	5.02 × 10^−5^	Lower	−5.74	3.06 × 10^−5^	1.69 × 10^−3^	Lower
84223	IQCG	−1.73	2.58 × 10^−8^	1.00 × 10^−7^	Lower	−2.68	1.31 × 10^−3^	1.31 × 10^−2^	Lower
51314	NME8	−2.38	5.98 × 10^−6^	1.36 × 10^−5^	Lower	−3.72	7.13 × 10^−5^	2.42 × 10^−3^	Lower
115948	ODAD3	−1.51	3.65 × 10^−2^	4.23 × 10^−2^	Lower	−2.56	5.91 × 10^−3^	3.40 × 10^−2^	Lower
84074	QRICH2	−1.41	1.52 × 10^−5^	3.09 × 10^−5^	Lower	−3.68	3.99 × 10^−5^	1.81 × 10^−3^	Lower
83853	ROPN1L	−2.11	1.41 × 10^−8^	6.00 × 10^−8^	Lower	−2.59	5.87 × 10^−3^	3.39 × 10^−2^	Lower
124404	SEPTIN12	−2.47	1.17 × 10^−11^	2.20 × 10^−10^	Lower	−2.88	2.21 × 10^−4^	4.66× 10^−3^	Lower
116369	SLC26A8	−1.69	1.13 × 10^−7^	3.93 × 10^−7^	Lower	−3.53	1.08 × 10^−6^	5.05 × 10^−4^	Lower
4184	SMCP	−1.06	9.29 × 10^−4^	1.31 × 10^−3^	Lower	−3.21	1.23 × 10^−3^	1.27 × 10^−2^	Lower
79925	SPEF2	−0.97	4.20 × 10^−4^	6.37 × 10^−4^	Lower	−2.77	2.19 × 10^−4^	4.66 × 10^−3^	Lower
27285	TEKT2	−1.11	7.57 × 10^−5^	1.33 × 10^−4^	Lower	−3.14	3.49 × 10^−5^	1.78 × 10^−3^	Lower
64518	TEKT3	−0.95	2.78 × 10^−3^	3.53 × 10^−3^	Lower	−3.08	4.86 × 10^−5^	2.01 × 10^−3^	Lower
122664	TPPP2	−1.82	8.67 × 10^−10^	6.36 × 10^−9^	Lower	−2.47	1.37 × 10^−5^	1.19 × 10^−3^	Lower
84203	TXNDC2	−1.61	3.34 × 10^−7^	9.38 × 10^−7^	Lower	−3.87	2.47 × 10^−5^	1.55 × 10^−3^	Lower

An unpaired two-tailed *t*-test was used to calculate the *p*-value. A false discovery rate (FDR) correction was used to adjust the *p*-values. An adjusted *p*-value < 0.05 was considered statistically significant.

**Table 4:** Significant correlations between significant shared genes and proteins with sperm motility.

Genes/Proteins	Motility (% Motile) (Genes Determined by RT-qPCR)		Motility (% Motile) (Proteins Determined by LC-MS/MS)	
	Correlation Coefficient (r)	*p*-Value	Correlation Coefficient (r)	*p*-Value
ATP1A4	0.18	1.69 × 10^−2^	0.61	2.69 × 10^−2^
CFAP157	0.21	1.29 × 10^−2^	0.66	1.42 × 10^−2^
CFAP206	0.32	1.56 × 10^−5^	0.61	2.69 × 10^−2^
CFAP251	0.18	1.46 × 10^−2^	0.66	1.42 × 10^−2^
CFAP43	0.26	4.42 × 10^−4^	0.59	3.25 × 10^−2^
CFAP44	0.16	3.10 × 10^−2^	0.62	2.52 × 10^−2^
CFAP65	0.22	1.32 × 10^−2^	0.65	1.53 × 10^−2^
DNAH1	0.35	1.79 × 10^−6^	0.62	2.52 × 10^−2^
DNAI1	0.26	5.46 × 10^−4^	0.60	2.87 × 10^−2^
DPCD	0.16	4.67 × 10^−2^	0.65	1.53 × 10^−2^
FSIP2	0.22	3.07 × 10^−3^	0.62	2.52 × 10^−2^
QRICH2	0.26	4.05 × 10^−4^	0.63	2.05 × 10^−2^
ROPN1L	0.31	1.71 × 10^−5^	0.74	4.11 × 10^−3^
SEPTIN12	0.38	9.71 × 10^−8^	0.80	9.69 × 10^−4^
SLC26A8	0.31	2.77 × 10^−5^	0.59	3.25 × 10^−2^
SPEF2	0.15	4.16 × 10^−2^	0.66	1.42 × 10^−2^
TEKT2	0.18	1.74 × 10^−2^	0.61	2.69 × 10^−2^
TPPP2	0.38	1.40 × 10^−7^	0.63	2.20 × 10^−2^
TXNDC2	0.26	4.83 × 10^−4^	0.71	6.68 × 10^−3^

## Data Availability

Proteomic data were obtained ProteomeXchange with the assigned accession number PXD039703 as indicated in Table S6.

## Supplementary Materials

The following supporting information can be downloaded at: https://www.mdpi.com/article/10.3390/cells12091239/s1: Figure S1: Volcano plot showing the differential expression levels of genes in sperm samples collected from oligoasthenozoospermic men (OA, n = 92) as compared to normozoospermic men (N, n = 92) as determined by RT-qPCR. Log2 (fold change) is plotted against the −log10 *p*-value. Figure S2: Heatmaps representing hierarchical clustering of the differentially higher-expressed genes (i.e., based Log2 (fold change) and adjusted *p*-value) in sperm samples of oligoasthenozoospermic men (OA, n = 92, light grey) as compared to normozoospermic men (N, n = 92, dark grey). The heatmaps were generated based on ΔCt and the rectangle represents the normalized expression level of the genes (the higher the value, the lower the expression level of genes). (Yellow, lower-expressed genes; Blue, higher-expressed genes). Figure S3: Heatmaps representing hierarchical clustering of the differentially lower-expressed genes (i.e., based on Log2 fold change and adjusted *p*-value) in sperm samples of oligoasthenozoospermic men (OA, n = 92, light grey) as compared to normozoospermic men (N, n = 92, dark grey). The heatmaps were generated based on ΔCt and the rectangle represents the normalized expression level of the genes (the higher the value, the lower the expression level of genes). Yellow, lower expressed genes; Blue, higher expressed genes. Figure S4: Heatmaps representing hierarchical clustering of all differentially higher-expressed proteins (i.e., based Log2 (fold change) and adjusted *p*-value) in sperm samples of oligoasthenozoospermic men (OA, n = 6, light grey) as compared to normozoospermic men (N, n = 7, dark grey). The heatmaps were generated based on log2 LFQ intensities and the rectangle represents the normalized expression level of the protein (the higher the value, the higher the expression level of protein). Yellow, higher-expressed proteins; Blue, lower-expressed proteins. Figure S5: Heatmaps representing hierarchical clustering of all differentially lower-expressed proteins (i.e., based Log2 (fold change) and adjusted *p*-value) in sperm samples of oligoasthenozoospermic men (OA, n = 6, light grey) as compared to normozoospermic men (N, n = 7, dark grey). The heatmaps were generated based on log2 LFQ intensities and the rectangle represents the normalized expression level of the protein (the higher the value, the higher the expression level of protein). Yellow, higher-expressed proteins; Blue, lower-expressed proteins. Table S1: The primers and design RefSeq of the genes associated with sperm motility. Table S2: Included samples obtained from ProteomeXchange with the assigned accession number PXD039703. Table S3: Significantly and differentially expressed proteins in the sperm samples of men with oligoasthenozoospermia compared to normozoospermic men as determined by LC-MS/MS. Table S4: List of significant shared genes and proteins in sperm samples of men with oligoasthenozoospermia compared to normozoospermic men as determined by RT-qPCR and LC-MS/MS. Table S5: Correlation of the expression level of significant shared genes and proteins as determined by RT-qPCR and LC-MS/MS with basic semen parameters. Table S6: Enriched Gene Ontology terms of adjusted significant proteins of oligoasthenozoospermic men in comparison to normozoospermic controls as determined by overrepresentation analysis.
