# Supplementary material for: Sperm Motility Annotated Genes: Are They Associated with Impaired Fecundity?
Source: Cells. 2023 Apr 25;12(9):1239. doi: 10.3390/cells12091239 (PMC10177407; doi:10.3390/cells12091239)
Supplement: Supplementary file 1 [file cells-12-01239-s001.zip › Supplementary Table 1.pdf]

Table S1: The primers and design RefSeq of the genes associated with sperm motility.

Gene	Gene ID	Reverse Primer	Reverse Primer	Design RefSeq
ADA (adenosine deaminase)	100	TACCTCACTGGTGCCTGGAA	TGTGTTGAGCGAGTAGTTAGCC	NM_000022.N
ADCY3 (adenylate cyclase 3)	109	ATGAAGGATACGCTCACCAACA	AGAACCCCGCCTTTGTTCA	NM_001320613.N
AKAP4 (A-kinase anchoring protein 4)	8852	ACAACCCAGAAGGACAGCAA	TCTTCTACATTCAGGGTGGACAC	NM_003886.N
ANXA5 (annexin A5)	308	CCTGAAGAACTGAGAGCCATCA	CCCACCACGTCATCTTCCA	NM_001154.N
APOB (apolipoprotein B)	338	AAGCCATCTGCAAGGAGCAA	AGTCTGTGTCACTTGTGCTACC	NM_000384.N
ARMC2 (armadillo repeat containing 2)	84071	ATGGAACAGTACAAGGGTGACAA	TGCAGCAGTCACGGTAAGAA	NM_032131.N
ASH1L (ASH1 like histone lysine methyltransferase)	55870	ATTGTAGCCCTACCCGGAAA	TCTTGGACTTCGAGAGGGAA	NM_001366177.N
ATP1A4 (ATPase Na+/K+ transporting subunit alpha 4)	480	GAATGACCTGGAGGACAGCTA	GGCCGTTTGGCATGTGAA	NM_144699.N
ATP2B4 (ATPase plasma membrane Ca2+ transporting 4)	493	AACGGTGGCTTCCGTATGTA	TTTCCGGTCCAGGATTCGATTA	NM_001001396.N
BBS2 (Bardet-Biedl syndrome 2)	583	GCCAGTGAGCCAATCAGTTA	AAAGTTCTGACCGAGCCATAC	NM_031885.N
HOATZ (HOATZ cilia and flagella associated protein)	399949	ACTCCTCGCAGTCTTTTCAC	ATCTTTAGGGCTTCGGCAAA	NM_207430.N
CACNA1E (calcium voltage-gated channel subunit alpha1 E)	777	TGTGGTCTGCCATCTACTTCA	CACAGCGATAGCCAAGAACA	NM_001205293.N
CACNA1I (calcium voltage-gated channel subunit alpha1 I)	8911	CGCTGGGTCCATCACAAATAC	TCCTTGGATGCCAGGACAAA	NM_021096.N
CATSPER1 (cation channel sperm associated 1)	117144	TGAAGCGGCTCATCGAGAAAA	CACCAGCTGCAGGTAATGGAA	NM_053054.N
CATSPER2 (cation channel sperm associated 2)	117155	AGCACTGACGTCAAGCCATA	AGTCCAAACTTTCCCTTTGTTGAC	NM_172095.N
CATSPER3 (cation channel sperm associated 3)	347732	AAGGTACCGCTTGTTCAGAC	CAGATGTGCAGATGGACACA	NM_178019.N
CATSPER4 (cation channel sperm associated 4)	378807	GCTCTCCGTACCAACTCCTA	GGATGGTCAGCACAATGTCA	NM_198137.N
CATSPERD (cation channel sperm associated auxiliary subunit delta)	257062	CCACCTTCTACGAGAACGGTTA	CCAGTGCTGCTGCTGTTTTA	NM_152784.N
CATSPERZ (catsper channel auxiliary subunit zeta)	25858	TGCCCCTGATGGAACTCA	CTGCCGATCCCTAACTGGTA	NM_001039496.N
ODAD3 (outer dynein arm docking complex subunit 3)	115948	GTGAACCAAGAGGCCCTCAA	TCTCGAACCAAGGTCTCCTCTA	NM_145045.N
CCDC39 (coiled-coil domain containing 39)	339829	GAGGAGAAAACACAGGCCTA	TCCAAACAGTCACCTTCCC	NM_181426.N
CCDC40 (coiled-coil domain containing 40)	55036	TCGGCTTCTGAGGGAGAGAA	TCACCTTTCTGGCCATCATCC	NM_017950.N
CCNYL1 (cyclin Y like 1)	151195	GCAAAGAAAGAGCACAGAACC	TCTCATAGCGGCTCTACACA	NM_001330218.N
CCR6 (C-C motif chemokine receptor 6)	1235	AGGCAGCGATGTCTGTGAA	AGCTCAAGCCCCAACATCA	NM_031409.N
CELF3 (CUGBP Elav-like family member 3)	11189	GCCATCAACACCCTTCACA	TCCTTCTCAGTGTCAGCAAAC	NM_001172648.N
CEP131 (centrosomal protein 131)	22994	CTACGAGGCCACCATCCA	TCGCACTTTTCACTCAGGAC	NM_001319228.N
CFAP157 (cilia and flagella associated protein 157)	286207	AAGAAGTCGGTGCTGGACAA	TTCGTGGTCACCTTGTGGAA	NR_145961.N
CFAP206 (cilia and flagella associated protein 206)	154313	AAAGATAGCGGTCCCAACATCA	GCAAGCTGGTCCACAGAGTA	NM_001031743.N
CFAP43 (cilia and flagella associated protein 43)	80217	TTCCAGGATTGACCAGAAGGAC	GGTAGGTGCCACAGTAACTGAA	NM_025145.N
CFAP44 (cilia and flagella associated protein 44)	55779	AAGGCCAAAGTTCCATCTCC	CTGTAGGCTTTGGGATCTTCAA	NM_001164496.N
CFAP65 (cilia and flagella associated protein 65)	255101	TGCCTGCAGCACCATCA	ACCTCAATGCCCCAGATGAC	NM_194302.N

CFAP69 (cilia and flagella associated protein 69)	79846	TGCTCGAGTCCTTGCATTTCTA	GGCCACCTGTACCATGAAAAC	NM_001039706.N
CFAP97D1 (CFAP97 domain containing 1)	284067	GAAACTGGACTTTGGCCACTAC	GCCACTGGAGGTTTGGTATCA	NM_001136483.N
CHRNA7 (cholinergic receptor nicotinic alpha 7 subunit)	1139	GATGGCCAGATTTGGAAACCA	CACCAACACGTTAGTGTGGAA	NM_001190455.N
DDX4 (DEAD-box helicase 4)	54514	CGTGTTTGCATCAGTTGATACC	CTGGATTGGGAGCTTGTGAA	NM_024415.N
DEFB1 (defensin beta 1)	1672	CTGCCAGTCGCCATGAGAA	GGCCAAGGCCTGTGAGAAA	NM_005218.N
DNAH1 (dynein axonemal heavy chain 1)	25981	GTCAGCGTGAATGACTTCCA	CAGCATTCACAGCACGGATA	NM_015512.N
DNAH11 (dynein axonemal heavy chain 11)	8701	TGCAAGGAGCTTCTCTCCAA	AGAGATCCAGCCACAACCAA	NM_001277115.N
DNAH5 (dynein axonemal heavy chain 5)	1767	TGACCCTTCCAAAGCCATCA	TCCATCTGCCGCATCTAACA	NM_001369.N
DNAI1 (dynein axonemal intermediate chain 1)	27019	GGGAAGATGGCCATGAGGAA	TGATCTTAGCAGCTTGGGACAA	NM_001281428.N
DNAJA1 (DnaJ heat shock protein family (Hsp40) member A1)	3301	CAACCGAACCATCGTCATCAC	GGCATGCCTTCATTTAGTACACA	NM_001539.N
DPCD (deleted in primary ciliary dyskinesia homolog (mouse))	25911	CTGCGGACAGCCCAGA	TTCCTTGCCGTCTGGGAAT	NM_001329746.N
DRC7 (dynein regulatory complex subunit 7)	84229	ACATGCAGGATTGCTGGAAC	GGAGCATGTACTCCCATCTCA	NM_001289162.N
EFCAB1 (EF-hand calcium binding domain 1)	79645	GAGGAAGACCCTGATGAAGGAA	GCAAAAGACAGCTTCCCATCA	NM_001142857.N
EFCAB9 (EF-hand calcium binding domain 9)	285588	GACGTGCACGGCAAGAA	CCAGTCCAGCATGTCAAACA	NM_001171183.N
ENKUR (enkurin, TRPC channel interacting protein)	219670	AGCCTTCAGGACTAGTTCCA	CTTTATTTCCTCGTTTCGCTTACA	NM_145010.N
ENO4 (enolase 4)	387712	CAACTCCACCTTCTACCTCCA	GTGGGAGGCTTTGCAAGTTTA	NM_001242699.N
EPPIN (epididymal peptidase inhibitor)	57119	CAAAGCCAACTGCCTGAACA	GCAGTTCTTCCAGTGCATCC	NM_001302861.N
FSIP2 (fibrous sheath interacting protein 2)	401024	ATTGGGATGGAAGACCAACC	ATATACCCGGGTCTGTAGCA	NM_173651.N
GAPDHS (glyceraldehyde-3-phosphate dehydrogenase, spermatogenic)	26330	GAGCTGACTGTGGGCATCA	CCTTAACACCCTTCTCCATGCA	NM_014364.N
GAS8 (growth arrest specific 8)	2622	ACCATCAAGGACCTGCAGTA	CCAGCAGCTTTGCCTCATA	NM_001286205.N
H1-6 (H1.6 linker histone, cluster member)	3010	CGCATCAAACTGTCCCTCAA	GCTTAAAGGAACCGGAAGCA	NM_005323.N
IFT88 (intraflagellar transport 88)	8100	CAGTGCCGAAGTTCTTTACCA	CCACCTGCATTAGCCATTCA	NR_134653.N
ING2 (inhibitor of growth family member 2)	3622	GTGGAAAATCGGGCAAGACAA	TCTGAGGCTCGTTCACTTTCA	NM_001564.N
INPP5B (inositol polyphosphate-5-phosphatase B)	3633	AGCCAATCTTGGACCTACCA	TATCCAACTGGCTCCCATCA	NR_158628.N
INSL6 (insulin like 6)	11172	CCGGGGAAGAGGCACAAA	CAGGTAGTGACTGCATTTCCC	NM_007179.N
INTS13 (integrator complex subunit 13)	55726	AGTAGACCTTCCTCCTGCCTTA	CCTGACTTTCGTGGTTGTTCC	NM_018164.N
IQCF1 (IQ motif containing F1)	132141	TGTCCTTAGGAGCAGAGTCAA	TGTCCACTGTCTGTGTCTCA	NM_152397.N
IQCG (IQ motif containing G)	84223	ACCATCATGACTACGGAGACAC	GGCCGAATCTTGCAACTCC	NM_001323027.N
KIAA0319L (KIAA0319 like)	79932	TCAGAGCCTTGGAAGTCAACA	CGTTTGGTGAACGAGTCACA	NM_024874.N
LDHC (lactate dehydrogenase C)	3948	ACAAGCTGCCATGGTTGGATTA	AGCAACATTCACCCCACTCC	NM_017448.N
LRRC6/DNAAF11 (dynein axonemal assembly factor 11)	23639	TGGTAGCAACTCTTCCACAA	GTCCTGCAATGCCTTAATCC	NM_001321965.N
LY6K (lymphocyte antigen 6 family member K)	54742	GAGACAACGAGATCCAGAGGAC	TTGGGTTCTGGCACTCGAAA	NM_017527.N

MEIG1 (meiosis/spermiogenesis associated 1)	644890	GCAGGATATCGGGATGAAACC	TATCCTGTCTCCGGCCAAC	NM_001080836.N
MET (MET proto-oncogene, receptor tyrosine kinase)	4233	TGCATGAAGCAGGAAGGAAC	AGGGAAGGAGTGGTACAACA	NM_001127500.N
NEURL1 (neuralized E3 ubiquitin protein ligase 1)	9148	CCTGCACGGGACCATCA	AGCAGGGGAGTGATGGGATA	NM_004210.N
NME8 (NME/NM23 family member 8)	51314	ACTGGGACCAAGAACTGTTGAA	GGCAAACTGTCCATCGCAAA	NM_016616.N
NPHP4 (nephrocystin 4)	261734	CTCAGCTAGCTTCAGCAGGAA	AGGCGGCTTCTCAAAACCA	NR_111987.N
NSUN7 (NOP2/Sun RNA methyltransferase family member 7)	79730	AGCTGTATCTTCCCAAGTACC	ATCTTGGAAATCATATAGCATCACAA	NM_024677.N
PGAM4 (phosphoglycerate mutase family member 4)	441531	GCCTCACCTCAGTGCAGAA	TGGCAGCCACATCTGATCAA	NM_001029891.N
PGK2 (phosphoglycerate kinase 2)	5232	TGTGGTCCTGAGAGCAACAA	TCCTAACGGCCCATTCCAAA	NM_138733.N
PIH1D3/DNAAF6 (dynein axonemal assembly factor 6)	139212	ACAATCCTTGACCTTCGTACTCC	GCTTTGGCACTGGTACATTCC	NM_001169154.N
PITHD1 (PITH domain containing 1)	57095	GCAGATGAAGAGCTTCTGTTTAA	GTGTGAGTCATCATCCTCTCC	NM_020362.N
PLA2G3 (phospholipase A2 group III)	50487	TGGGCACAACCTGCTTCAA	GGCCCTAGGGTCTCTGGAA	NM_015715.N
PLTP (phospholipid transfer protein)	5360	ATCTGCCCTGTCCTCTACCA	CAACAAGCTCGTCCACAGAAC	NM_182676.N
PRDM14 (PR/SET domain 14)	63978	CACTCTGGAGACAGACCATACC	GAGTATGCTGGAGGCTGTGAA	NM_024504.N
PRM3 (protamine 3)	58531	CAACGAAGGCCCAGGAAG	TGACTCTTCTCTGAACACTGTC	NM_021247.N
PRSS55 (serine protease 55)	203074	ACTGCGGCTCACTGCTTATA	TGGCGACCTCCTTTATTTCCA	NM_198464.N
QRICH2 (glutamine rich 2)	84074	TGTCTCTGAAGCCTCCCTTTAC	GGAGGTCAGCATGCTCTCA	NM_032134.N
RABL2A (RAB, member of RAS oncogene family like 2A)	11159	CAGCAGCTGTCCACGTA	AGGATGGTCTTGCCATCTAC	NM_001354409.N
RABL2B (RAB, member of RAS oncogene family like 2B)	11158	ACAGCCACGGTAGATGGAA	GTGGTAGTAGGAGGCATGCA	NM_001350004.N
RGN (regucalcin)	9104	GGCCTGTTACAATGGAGGAA	CCCTCCAAAGCAGCATGAA	NM_001282848.N
RNASE10 (ribonuclease A family member 10 (inactive))	338879	AGCCCAGTATGCATTCATCCA	TGAGCTCACAGGCAATGACA	NM_001012975.N
RNASE9 (ribonuclease A family member 9 (inactive))	390443	GGAGCCACCTGAACACAGAA	CCTCCCACCCTAAGCTCTCA	NM_001110359.N
ROPN1 (rhophilin associated tail protein 1)	54763	CCAACCAATCAATGGCTCAGAC	GGCTTTGGCAAACTCCTTCA	NR_133918.N
ROPN1B (rhophilin associated tail protein 1B)	152015	TGTAACTGGGCAGAGCTAACA	GTTGGGAGATTCACCACTTTCC	NM_001308313.N
ROPN1L (rhophilin associated tail protein 1 like)	83853	CCCTTGGAGACGGAATCCTAC	AGACCTATCATGCCGTTCTTCC	NM_031916.N
SEMG1 (semenogelin 1)	6406	GCTCCTCATCTTGGAGAAGCA	CTTGGTAATCGGCCTTTTGATCC	NM_003007.N
SEMG2 (semenogelin 2)	6407	TGTCCTTTCCCTGCTCCTTA	GGCAATTGGCCTTTTGATCC	NM_003008.N
SEPTIN12 (septin 12)	124404	AAGACCAAGTGGGGCATCA	CAGGTCTCTCAGGAGAGGAAA	NM_144605.N
SLC22A14 (solute carrier family 22 member 14)	9389	ACTTGTGATCCCCTTCATCTCC	CCTCCTTCACCTTCCCTTTCA	NM_004803.N
SLC22A16 (solute carrier family 22 member 16)	85413	GGATCGGTGACTTTTGGCTAC	GCCGCTGCTATTCCAAACA	NM_033125.N
SLC26A8 (solute carrier family 26 member 8)	116369	TAAGCATGGCCACAGAAACC	GAAATCTGGTGTTACAGGAAGCA	NM_001193476.N
SLC9B1 (solute carrier family 9 member B1)	150159	CATGCTTGAGCATAGTCTTTTCC	CAATTCCTGCCAGCAGACTA	NM_139173.N
SLC9B2 (solute carrier family 9 member B2)	133308	ACGTTGGTCATGGCTTTCC	GTCCCAGGCAACTGCAATTA	NM_178833.N
SLC9C1 (solute carrier family 9 member C1)	285335	AGTACTGAGGACCTCCCTGAA	CTTCCAAGTGCCGGTTCAAA	NM_183061.N

SLIRP (SRA stem-loop interacting RNA binding protein)	81892	CGGAATGCACTACAACAGGAA	GCGGAAGTTTTGGCCTTCTA	NR_052025.N
SMCP (sperm mitochondria associated cysteine rich protein)	4184	GAGAAGTCAAGCATGGAAATTGT	CACACATCTTCTGAACACTTGG	NM_030663.N
SORD (sorbitol dehydrogenase)	6652	CACTTTGCTCGTGGCCAAA	TGGCTTTGGACAATCGGGTA	NR_034039.N
SPAG16 (sperm associated antigen 16)	79582	GCAATTCAGATGGCCCAAGAA	GGTACTGCATGCTTTGAAGGAA	NR_047659.N
SPAG6 (sperm associated antigen 6)	9576	CCTAGCAATGGCAGTCATCA	AAGCAGCTGCAGCCTTAATA	NM_001253854.N
SPEF2 (sperm flagellar 2)	79925	CACGAGAGCAAAGGGAGAAA	ATCAGCTGTTCCTCCCGATA	NM_024867.N
SPEM1 (spermatid maturation 1)	374768	TGGTGTCTTATACCAAGTGTTCCA	GCTGGGTCTGCTTTCTGAGTA	NM_199339.N
TAC1 (tachykinin precursor 1)	6863	TCCACTCAGCTGTTTGCAGAA	GCTGAGGCTTGGGTCTCC	NM_003182.N
TAC3 (tachykinin precursor 3)	6866	AGTTCCTTGATCCTGCCAGAC	GCTGTGAATAGCAGCATGATCC	NR_135164.N
TAC4 (tachykinin precursor 4)	255061	TCAGCACTGAAGCAGAGACC	TTGCCTTGCCCGTCTTCA	NM_170685.N
TACR1 (tachykinin receptor 1)	6869	GACTCCTCTGACCGCTACCA	TGCACACCACGACAATCATCA	NM_001058.N
TACR2 (tachykinin receptor 2)	6865	CTGGTTGGCCATGAGCTCTA	AAGGCAAGCCGGAATCCA	NM_001057.N
TACR3 (tachykinin receptor 3)	6870	TGTTCGCCAGCATCTACTCC	CAGTCTGGGTTTCAAGGGATCA	NM_001059.N
TAF7L (TATA-box binding protein associated factor 7 like)	54457	TCGGATGCTGAAGCCGTAA	GGGATGGAGCCTTGACTTTCTA	NM_024885.N
TCTE1 (t-complex-associated-testis-expressed 1)	202500	ACCACACTCACCAGCATCAA	TGTTGTCTGACATGCCTTCCA	NM_182539.N
TEKT2 (tektin 2)	27285	ACCAAGAAGGCCTTGCAACA	TTGAGCTGCTGTTGGACTTCC	NM_014466.N
TEKT3 (tektin 3)	64518	TGGATAAGGCTATTGCCCAAC	GCCGTCTGTTTGTCACTCA	NM_031898.N
TEX101 (testis expressed 101)	83639	CCTGATCGTGACCTCCTACA	GACAATGGAGGGTTGTTGACA	NM_031451.N
TMF1 (TATA element modulatory factor 1)	7110	GAAGGTGAAGGAGATACCCAAA	CATCTGCAGAATAGTGTTGTACC	NM_001363879.N
TMPRSS12 (transmembrane serine protease 12)	283471	CTGCCCACTGCACTAAAGAC	GGTATGAGGATAGCGTCCATGTA	NM_182559.N
TNP1 (transition protein 1)	7141	TACCGTAAGGGCAACCTGAA	TCACAAGTGGGAGCGGTAA	NM_003284.N
TNP2 (transition protein 2)	7142	AGCAGGTGTACAAAACCAAGAC	GGTGAAACACGCAGGAACA	NM_005425.N
TPGS1 (tubulin polyglutamylase complex subunit 1)	91978	CGCGCTATGGCACCTT	GCTCACGTTGTTGTTGAAGG	NM_033513.N
TPPP2 (tubulin polymerization promoting protein family member 2)	122664	GTGGACATCGTGTTCAGCAA	TTCTGGCCCAGTTCCTTCA	NM_173846.N
TSSK4 (testis specific serine kinase 4)	283629	AGGTCACTTTCCCAGCTAACC	TTAGTGGCTTGGCGTAGCA	NM_001184739.N
TTC12 (tetratricopeptide repeat domain 12)	54970	ACCTGGCCCTGAAGAACTAC	GGTTTGCAGCTTGGGGTTTA	NM_001352038.N
TTC21A (tetratricopeptide repeat domain 21A)	199223	TTTGAAGCAGGCACTGGAAC	CCAGCAAAAGCAGGCACTTA	NM_145755.N
TTLL1 (tubulin tyrosine ligase like 1)	25809	ATGGAACAAGTCGCCACCTA	ACCCTGGGCCAATTCTTCA	NR_027779.N
TTLL5 (tubulin tyrosine ligase like 5)	23093	ATGCGCCTCTGGACCTAAA	GCAGGATCTTGGCACACAAA	NM_015072.N
TTLL9 (tubulin tyrosine ligase like 9)	164395	GTGGCTCAGCGTTACATTGAA	GACATCACCAGCACATAGACAC	NR_148011.N
TXNDC2 (thioredoxin domain containing 2)	84203	TTCCATGCCCTGTCTGTGAA	ACTCTCTCACCACCTCCTCA	NM_001098529.N
UBE2B (ubiquitin conjugating enzyme E2 B)	7320	GGAGTCCAACATATGATGTATCTTCTA	TGGACTGTTAGGATTCGGTTCA	NM_003337.N
VPS13A (vacuolar protein sorting 13 homolog A)	23230	CACAGGCACTGCTGTAGAAAC	CCCACTTCACTGATTCCTGTGTA	NM_033305.N

WDR66/CFAP251 (cilia and flagella associated protein 251)	144406	GCTTTTCCCATGACTCCCAGTA	TCTGACCACCAGCATGTAAACA	NM_144668.N
WT1 (WT1 transcription factor)	7490	ACCAGCTCAAAAGACACCAAA	GGGAGAACTTTCGCTGACAA	NR_160306.N
ZMYND10 (zinc finger MYND-type containing 10)	51364	ATTCAGGAGCTGCTGGTCAC	CCACATCTCCACTGCGATCA	NM_015896.N
**ACTB (actin beta)**	**60**	**CCAACCGCGAGAAGATGAC**	**TAGCACAGCCTGGATAGCAA**	**NM_001101.N**
**GAPDH (glyceraldehyde-3-phosphate dehydrogenase)**	**2597**	**GAGAACGGGAAGCTTGTCATCA**	**TGGACTCCACGACGTACTCA**	**NM_002046.**
