# Supplementary material for: Sperm Motility Annotated Genes: Are They Associated with Impaired Fecundity?
Source: Cells. 2023 Apr 25;12(9):1239. doi: 10.3390/cells12091239 (PMC10177407; doi:10.3390/cells12091239)
Supplement: Supplementary file 1 [file cells-12-01239-s001.zip › Supplementary Table 4.pdf]

Table S4: List of significant shared genes and proteins in sperm samples of men with oligoasthenozoospermia compared to normozoospermic men as determined by RT-qPCR and LC-MS/MS.

Entrez ID	Gene symbol	Genes (RT-qPCR)				Proteins (LC-MS/MS)			
		Log2 [fold change]	*p*-value	Adjusted *p*-value	Regulation	Log2 [fold change]	*p*-value	Adjusted *p*-value	Regulation
8852	AKAP4	-1.18	7.05E-04	1.01E-03	Lower	-1.57	1.77E-02	6.98E-02	None
308	ANXA5	-2.66	2.22E-10	2.25E-09	Lower	1.67	5.79E-03	3.39E-02	Higher
480	ATP1A4	-1.14	7.99E-04	1.13E-03	Lower	-2.65	7.63E-05	2.51E-03	Lower
493	ATP2B4	-0.08	6.72E-01	6.82E-01	None	-2.81	2.63E-05	1.56E-03	Lower
583	BBS2	1.78	5.21E-08	1.91E-07	Higher	-0.59	4.15E-01	5.17E-01	None
117155	CATSPER2	-1.59	2.47E-05	4.66E-05	Lower	-1.00	7.83E-02	1.70E-01	None
257062	CATSPERD	-1.48	2.84E-05	5.18E-05	Lower	-0.98	9.43E-02	1.92E-01	None
339829	CCDC39	-0.70	3.36E-02	3.96E-02	Lower	-2.72	1.33E-03	1.31E-02	Lower
55036	CCDC40	-0.16	4.29E-01	4.43E-01	None	-3.70	2.04E-06	5.05E-04	Lower
151195	CCNYL1	-1.82	1.48E-10	1.63E-09	Lower	0.81	4.30E-02	1.19E-01	None
286207	CFAP157	-1.57	2.77E-04	4.30E-04	Lower	-3.05	3.88E-05	1.81E-03	Lower
154313	CFAP206	-2.19	7.53E-10	5.85E-09	Lower	-3.95	1.14E-04	3.14E-03	Lower
144406	CFAP251	-1.21	1.26E-04	2.09E-04	Lower	-3.38	4.85E-06	6.99E-04	Lower
80217	CFAP43	-1.55	5.90E-07	1.59E-06	Lower	-2.83	1.41E-04	3.62E-03	Lower
55779	CFAP44	-1.35	4.11E-06	9.86E-06	Lower	-3.22	2.40E-05	1.55E-03	Lower
255101	CFAP65	-0.76	2.72E-02	3.24E-02	Lower	-2.90	5.73E-06	7.60E-04	Lower
79846	CFAP69	-2.44	4.88E-08	1.84E-07	Lower	-2.47	1.33E-03	1.31E-02	Lower
25981	DNAH1	-2.46	1.35E-09	8.85E-09	Lower	-4.14	7.19E-07	5.05E-04	Lower
27019	DNAI1	-1.57	1.08E-05	2.30E-05	Lower	-3.18	6.63E-04	8.63E-03	Lower
3301	DNAJA1	0.08	4.05E-01	4.21E-01	None	1.44	3.08E-02	9.89E-02	None
25911	DPCD	-1.73	2.37E-05	4.60E-05	Lower	-2.42	1.30E-05	1.16E-03	Lower
84229	DRC7	-0.82	1.37E-03	1.87E-03	Lower	-2.20	2.11E-03	1.76E-02	Lower
219670	ENKUR	-1.28	1.86E-08	7.45E-08	Lower	-2.94	3.09E-03	2.28E-02	Lower
401024	FSIP2	-1.50	2.70E-05	5.02E-05	Lower	-5.74	3.06E-05	1.69E-03	Lower
26330	GAPDHS	-1.48	8.77E-07	2.32E-06	Lower	-1.43	1.80E-02	7.05E-02	None
2622	GAS8	0.29	1.81E-01	1.98E-01	None	-3.37	6.03E-04	8.26E-03	Lower
3010	H1-6	0.77	9.96E-04	1.38E-03	Higher	-1.90	1.98E-01	3.06E-01	None
132141	IQCF1	-2.37	2.93E-07	8.60E-07	Lower	-2.57	2.63E-02	9.03E-02	None
84223	IQCG	-1.73	2.58E-08	1.00E-07	Lower	-2.68	1.31E-03	1.31E-02	Lower

79932	KIAA0319L	-0.52	8.05E-02	9.24E-02	None	0.87	5.84E-02	1.43E-01	None
3948	LDHC	-2.42	3.66E-10	3.22E-09	Lower	-0.96	1.29E-02	5.75E-02	None
51314	NME8	-2.38	5.98E-06	1.36E-05	Lower	-3.72	7.13E-05	2.42E-03	Lower
115948	ODAD3	-1.51	3.65E-02	4.23E-02	Lower	-2.56	5.91E-03	3.40E-02	Lower
5232	PGK2	-1.63	4.26E-03	5.35E-03	Lower	-1.05	6.60E-02	1.53E-01	None
57095	PITHD1	-0.05	6.88E-01	6.93E-01	None	-0.36	6.97E-01	7.68E-01	None
84074	QRICH2	-1.41	1.52E-05	3.09E-05	Lower	-3.68	3.99E-05	1.81E-03	Lower
54763	ROPN1	-1.51	1.56E-08	6.43E-08	Lower	-1.52	2.97E-02	9.67E-02	None
152015	ROPN1B	-2.01	1.69E-09	1.01E-08	Lower	-0.30	8.25E-01	8.71E-01	None
83853	ROPN1L	-2.11	1.41E-08	6.00E-08	Lower	-2.59	5.87E-03	3.39E-02	Lower
6406	SEMG1	-0.82	2.03E-04	3.20E-04	Lower	0.59	4.88E-01	5.83E-01	None
6407	SEMG2	-1.50	2.86E-05	5.18E-05	Lower	1.36	1.31E-01	2.35E-01	None
124404	SEPTIN12	-2.47	1.17E-11	2.20E-10	Lower	-2.88	2.21E-04	4.66E-03	Lower
116369	SLC26A8	-1.69	1.13E-07	3.93E-07	Lower	-3.53	1.08E-06	5.05E-04	Lower
150159	SLC9B1	-1.96	1.71E-12	5.65E-11	Lower	-1.05	2.61E-01	3.70E-01	None
4184	SMCP	-1.06	9.29E-04	1.31E-03	Lower	-3.21	1.23E-03	1.27E-02	Lower
6652	SORD	0.45	5.32E-04	7.89E-04	Higher	1.92	1.23E-02	5.56E-02	None
79582	SPAG16	-1.99	8.52E-09	3.75E-08	Lower	-1.38	3.78E-02	1.11E-01	None
9576	SPAG6	-1.92	6.59E-09	3.34E-08	Lower	-1.56	1.39E-01	2.43E-01	None
79925	SPEF2	-0.97	4.20E-04	6.37E-04	Lower	-2.77	2.19E-04	4.66E-03	Lower
374768	SPEM1	-1.43	4.40E-06	1.04E-05	Lower	-2.24	3.54E-02	1.08E-01	None
27285	TEKT2	-1.11	7.57E-05	1.33E-04	Lower	-3.14	3.49E-05	1.78E-03	Lower
64518	TEKT3	-0.95	2.78E-03	3.53E-03	Lower	-3.08	4.86E-05	2.01E-03	Lower
83639	TEX101	-1.79	1.72E-05	3.43E-05	Lower	-2.01	2.15E-02	7.95E-02	None
122664	TPPP2	-1.82	8.67E-10	6.36E-09	Lower	-2.47	1.37E-05	1.19E-03	Lower
54970	TTC12	-0.40	3.06E-01	3.25E-01	None	-1.63	4.64E-02	1.24E-01	None
199223	TTC21A	-1.69	9.47E-10	6.58E-09	Lower	-1.26	8.40E-02	1.78E-01	None
84203	TXNDC2	-1.61	3.34E-07	9.38E-07	Lower	-3.87	2.47E-05	1.55E-03	Lower
23230	VPS13A	-0.42	1.81E-01	1.98E-01	None	-2.65	1.74E-03	1.56E-02	Lower

- An unpaired two-tailed t-test was used to calculate the p-value.
- False Discovery Rate (FDR) correction was used to adjust the p-values.
- An adjusted p-value < 0.05 was considered statistically significant.
