# Supplementary material for: Sperm Motility Annotated Genes: Are They Associated with Impaired Fecundity?
Source: Cells. 2023 Apr 25;12(9):1239. doi: 10.3390/cells12091239 (PMC10177407; doi:10.3390/cells12091239)
Supplement: Supplementary file 1 [file cells-12-01239-s001.zip › Supplementary Table 5.pdf]

Table S5: Correlation of the expression level of significant shared genes and proteins as deter-mined by RT-qPCR and LC-MS/MS with basic semen parameters.

Gene RT-qPCR	Count (10⁶/mL)		Motility (% motile)		Morphology (%)		Protein LC-MS/MS	Count (10⁶/mL)		Motility (% motile)		Morphology (%)	
	r	*p*-value	r	*p*-value	r	*p*-value		r	*p*-value	r	*p*-value	r	*p*-value
ANXA5	0.42	<0.0001	0.34	<0.0001	0.25	0.0008	**ANXA5**	**-0.70**	**0.0082**	**-0.48**	**0.0941**	**-0.54**	**0.0548**
ATP1A4	0.20	0.0085	0.18	0.0169	0.18	0.0176	ATP1A4	0.71	0.0063	0.61	0.0269	0.53	0.0624
CCDC39	0.14	0.0821	0.13	0.0930	0.23	0.0028	CCDC39	0.77	0.0019	0.53	0.0640	0.59	0.0336
CFAP157	0.25	0.0027	0.21	0.0129	0.17	0.0420	CFAP157	0.80	0.0012	0.66	0.0142	0.66	0.0146
CFAP206	0.37	<0.0001	0.32	<0.0001	0.19	0.0111	CFAP206	0.78	0.0017	0.61	0.0269	0.52	0.0664
CFAP251	0.20	0.0081	0.18	0.0146	0.18	0.0188	CFAP251	0.92	<0.0001	0.66	0.0142	0.49	0.0892
CFAP43	0.26	0.0005	0.26	0.0004	0.19	0.0121	CFAP43	0.79	0.0014	0.59	0.0325	0.66	0.0146
CFAP44	0.16	0.0320	0.16	0.0310	0.19	0.0094	CFAP44	0.72	0.0054	0.62	0.0252	0.58	0.0362
CFAP65	0.15	0.0894	0.22	0.0132	0.11	0.2150	CFAP65	0.89	<0.0001	0.65	0.0153	0.46	0.1170
CFAP69	0.30	<0.0001	0.24	0.0014	0.16	0.0347	CFAP69	0.47	0.1024	0.55	0.0518	0.57	0.0418
DNAH1	0.44	<0.0001	0.35	<0.0001	0.19	0.0109	DNAH1	0.70	0.0082	0.62	0.0252	0.52	0.0706
DNAI1	0.28	0.0002	0.26	0.0005	0.19	0.0112	DNAI1	0.72	0.0052	0.60	0.0287	0.54	0.0548
DPCD	0.25	0.0026	0.16	0.0467	0.14	0.0893	DPCD	0.68	0.0102	0.65	0.0153	0.34	0.2523
DRC7	0.18	0.0152	0.23	0.0025	0.19	0.0097	DRC7	0.68	0.0106	0.37	0.2159	0.56	0.0480
ENKUR	0.39	<0.0001	0.29	0.0001	0.18	0.0156	ENKUR	0.62	0.0249	0.43	0.1383	0.64	0.0175
FSIP2	0.21	0.0066	0.22	0.0031	0.20	0.0095	FSIP2	0.78	0.0016	0.62	0.0252	0.54	0.0585
IQCG	0.27	0.0002	0.25	0.0007	0.22	0.0033	IQCG	0.72	0.0054	0.52	0.0673	0.74	0.0035
NME8	0.21	0.0117	0.15	0.0734	0.20	0.0169	NME8	0.75	0.0031	0.61	0.0269	0.56	0.0448
ODAD3	0.36	0.0040	0.03	0.8184	0.11	0.4177	ODAD3	0.74	0.0038	0.50	0.0819	0.60	0.0311
QRICH2	0.29	0.0001	0.26	0.0004	0.21	0.0040	QRICH2	0.65	0.0163	0.63	0.0205	0.51	0.0750
ROPN1L	0.32	<0.0001	0.31	<0.0001	0.25	0.0008	ROPN1L	0.72	0.0054	0.74	0.0041	0.38	0.1971
SEPTIN12	0.41	<0.0001	0.38	<0.0001	0.25	0.0006	SEPTIN12	0.80	0.0012	0.80	0.0010	0.35	0.2425
SLC26A8	0.31	<0.0001	0.31	<0.0001	0.22	0.0034	SLC26A8	0.79	0.0014	0.59	0.0325	0.63	0.0208
SMCP	0.17	0.0251	0.15	0.0400	0.11	0.1339	SMCP	0.60	0.0293	0.42	0.1497	0.64	0.0175
SPEF2	0.19	0.0093	0.15	0.0416	0.06	0.4339	SPEF2	0.79	0.0014	0.66	0.0142	0.49	0.0892
TEKT2	0.22	0.0024	0.18	0.0174	0.10	0.1779	TEKT2	0.76	0.0026	0.61	0.0269	0.54	0.0585
TEKT3	0.16	0.0272	0.14	0.0540	0.17	0.0237	TEKT3	0.83	0.0005	0.76	0.0024	0.30	0.3274
TPPP2	0.39	<0.0001	0.38	<0.0001	0.17	0.0237	TPPP2	0.82	0.0007	0.63	0.0220	0.44	0.1362
TXNDC2	0.27	0.0003	0.26	0.0005	0.19	0.0130	TXNDC2	0.64	0.0189	0.71	0.0067	0.50	0.0795

- Spearman correlation analysis was used.
- An Unpaired two-tailed t-test was used to calculate the *p*-value.
- $p < 0.05$ was considered statistically significant.
