# Supplementary figures and images for: Sperm Motility Annotated Genes: Are They Associated with Impaired Fecundity?
Source: Cells. 2023 Apr 25;12(9):1239. doi: 10.3390/cells12091239 (PMC10177407; doi:10.3390/cells12091239)

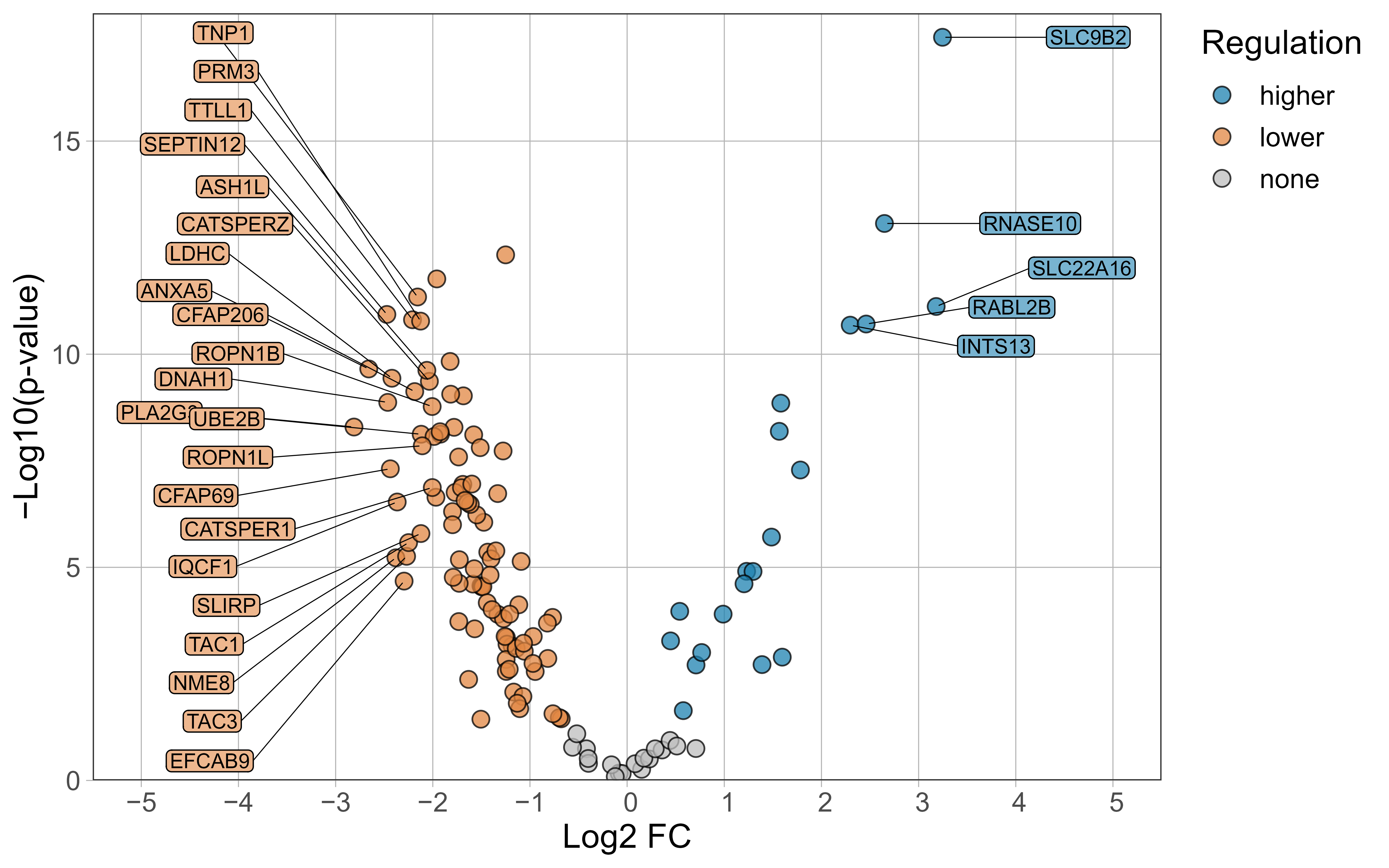

Supplement: Supplementary file 1 [file cells-12-01239-s001.zip › Supplementary Figure 1.png]

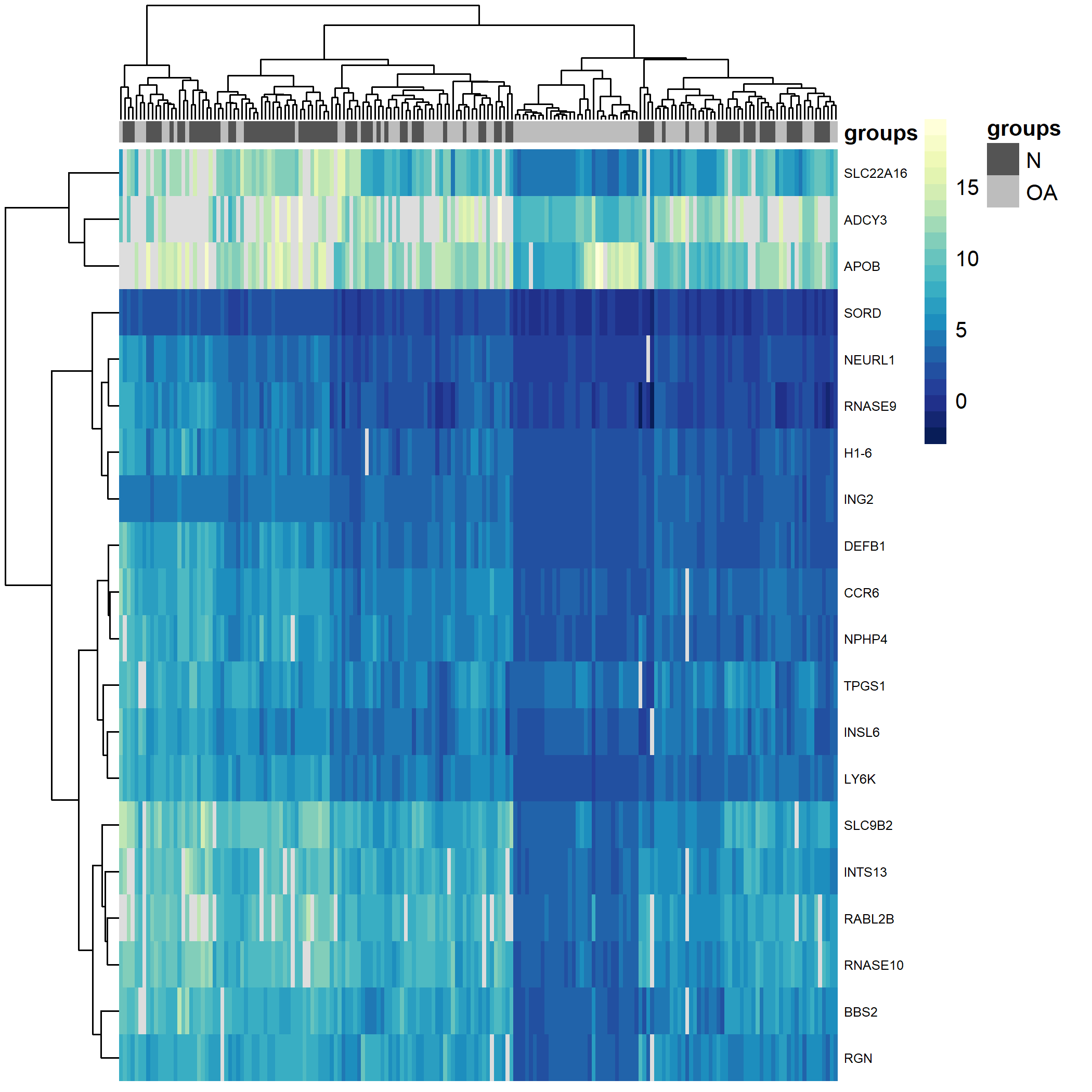

Supplement: Supplementary file 1 [file cells-12-01239-s001.zip › Supplementary Figure 2.png]

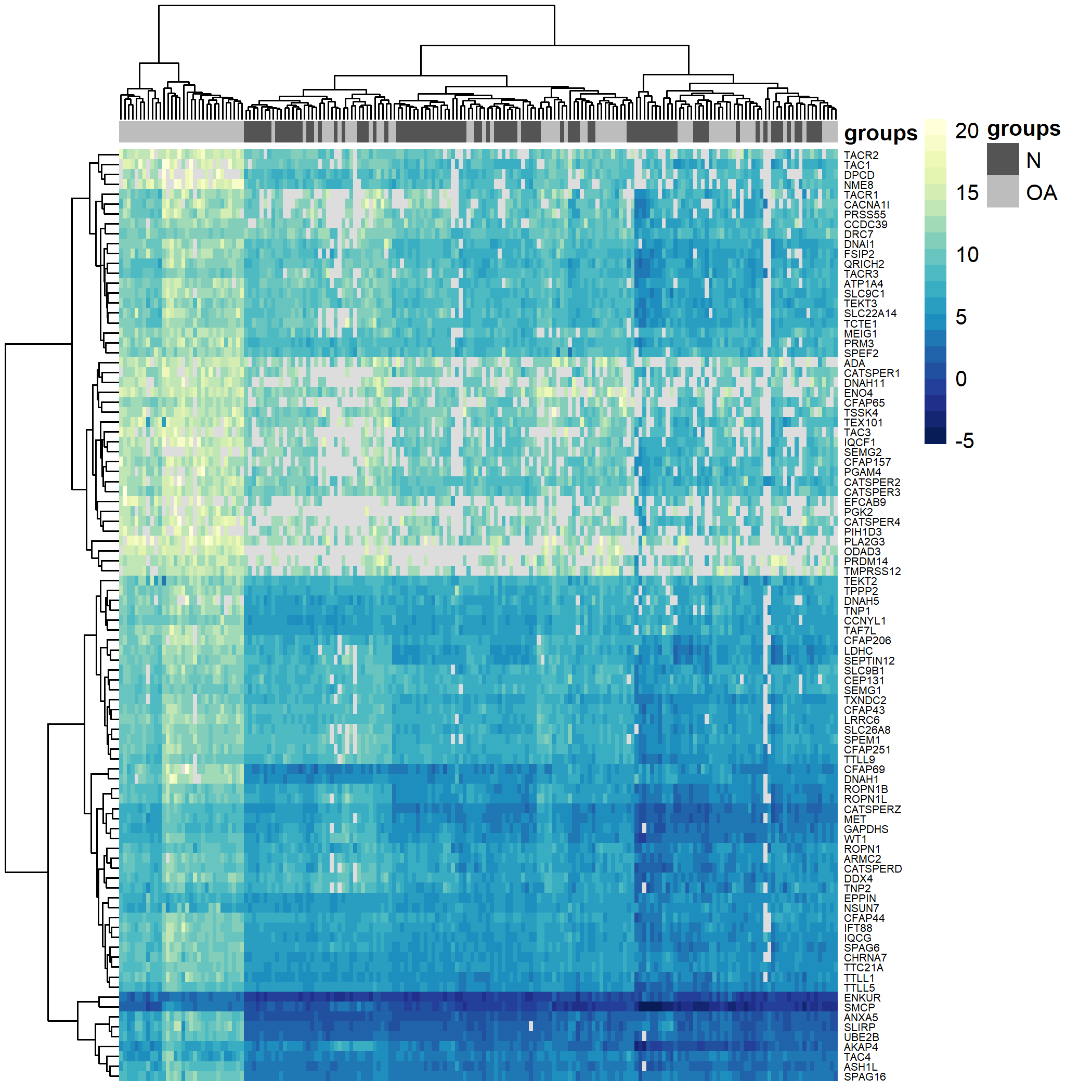

Supplement: Supplementary file 1 [file cells-12-01239-s001.zip › Supplementary Figure 3.png]

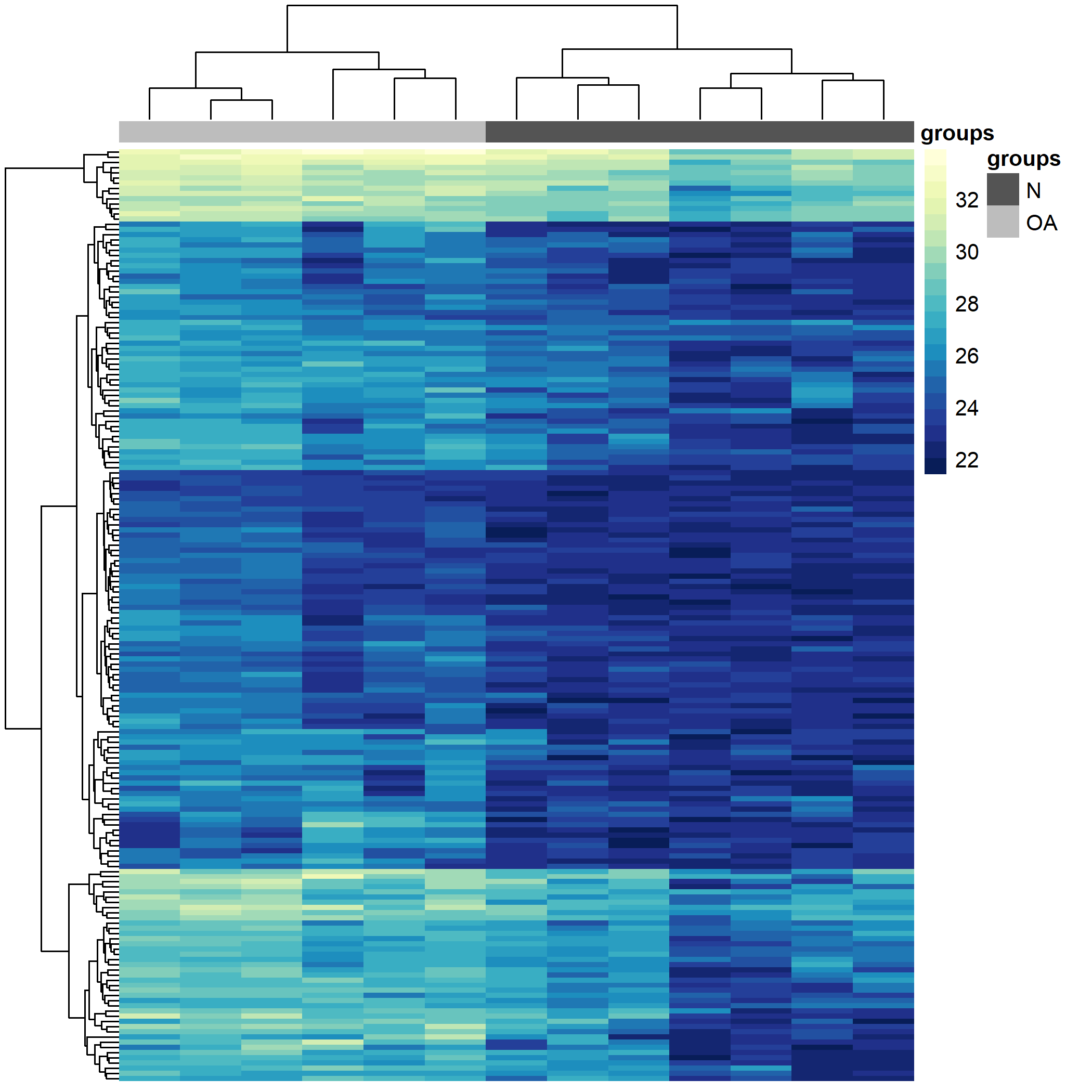

Supplement: Supplementary file 1 [file cells-12-01239-s001.zip › Supplementary Figure 4.png]

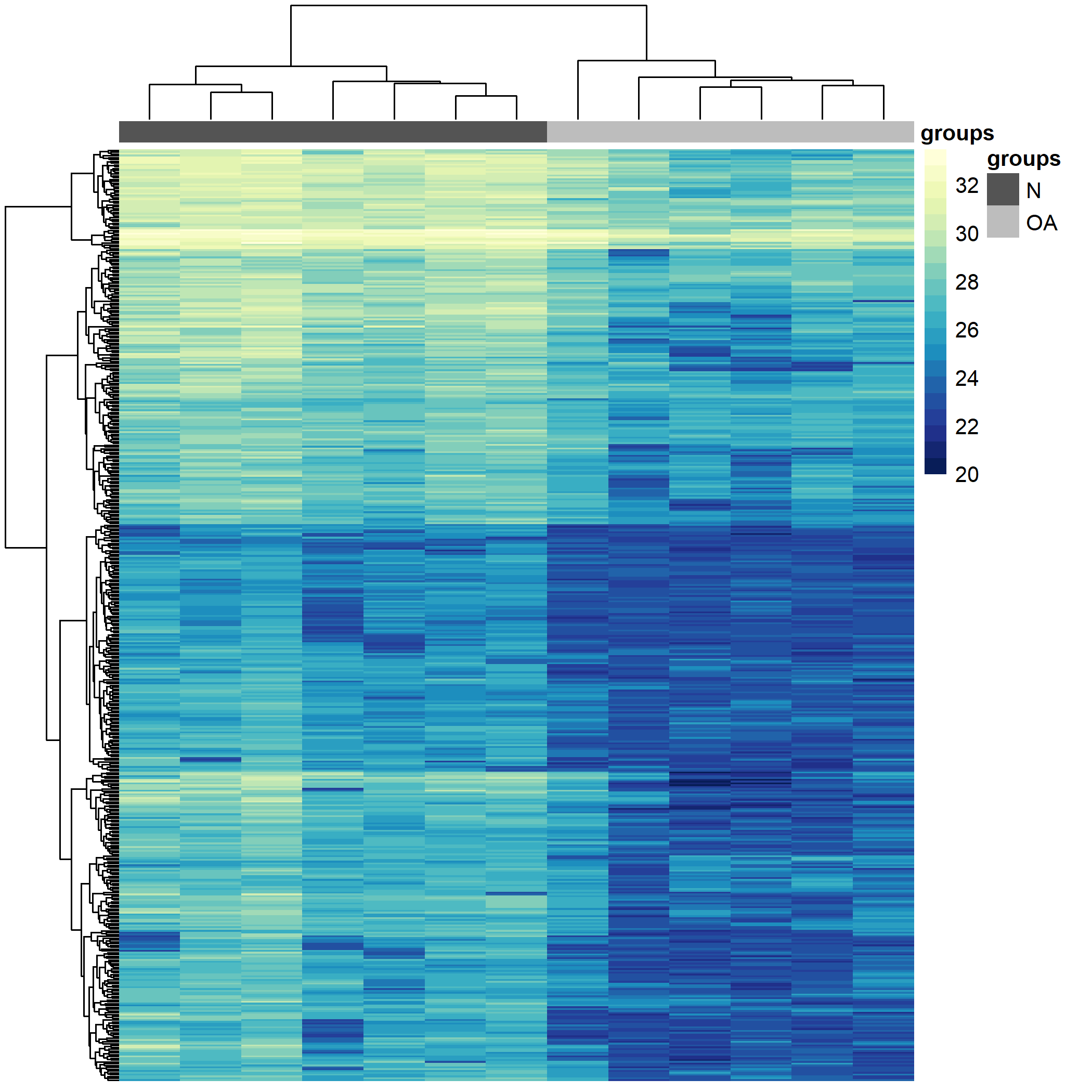

Supplement: Supplementary file 1 [file cells-12-01239-s001.zip › Supplementary Figure 5.png]
